# Kombucha Beverage from Green, Black and Rooibos Teas: A Comparative Study Looking at Microbiology, Chemistry and Antioxidant Activity

**DOI:** 10.3390/nu11010001

**Published:** 2018-12-20

**Authors:** Francesca Gaggìa, Loredana Baffoni, Michele Galiano, Dennis Sandris Nielsen, Rasmus Riemer Jakobsen, Josue Leonardo Castro-Mejía, Sara Bosi, Francesca Truzzi, Federica Musumeci, Giovanni Dinelli, Diana Di Gioia

**Affiliations:** 1 Department of Agricultural and Food Sciences (DISTAL), Alma Mater Studiorum-Università di Bologna, viale Fanin 42, 40127 Bologna, Italy; francesca.gaggia@unibo.it (F.G.); loredana.baffoni@unibo.it (L.B.); m.galiano@unibo.it (M.G.); sara.bosi@unibo.it (S.B.); francesca.truzzi3@unibo.it (F.T.); federica.musumeci2@unibo.it (F.M.); giovanni.dinelli@unibo.it (G.D.); 2 Department of Food Science, Faculty of Science, University of Copenhagen, Rolighedsvej 26, 1958 Frederiksberg C, Denmark; dn@food.ku.dk (D.S.N.); rasmus@food.ku.dk (R.R.J.); jcame@food.ku.dk (J.L.C.-M.)

**Keywords:** kombucha, rooibos, fermentation, oxidative stress

## Abstract

Kombucha is usually obtained from the fermentation of black or green tea by a consortium of acetic acid bacteria and yeasts. In this study, kombucha was prepared from the same starter consortium using green and black teas as well as, for the first time, an infusion of rooibos leaves (*Aspalathus linearis*). Microbial diversity was analysed during fermentation both in the biofilm and in the corresponding kombuchas, using culture-dependent and -independent methods. Polyphenols, flavonoids, ethanol, and acids were quantified and anti-oxidant activities were monitored. All of the Kombuchas showed similarity in bacterial composition, with the dominance of *Komagataeibacter* spp. Beta diversity showed that the yeast community was significantly different among all tea substrates, between 7 and 14 days of fermentation and between biofilm and kombucha, indicating the influence of the substrate on the fermenting microbiota. Kombucha from rooibos has a low ethanol concentration (1.1 mg/mL), and a glucuronic acid amount that was comparable to black tea. Although antioxidant activity was higher in black and green kombucha compared to rooibos, the latter showed an important effect on the recovery of oxidative damage on fibroblast cell lines against oxidative stress. These results make rooibos leaves interesting for the preparation of a fermented beverage with health benefits.

## 1. Introduction

Kombucha is a traditional beverage usually obtained from the fermentation of black or green tea (sweetened with 5–8% of sugar) by a symbiotic microbial consortium, which is mainly composed of acetic acid bacteria (AAB) and osmophilic yeasts [1]. The peculiarity is that the microorganisms are embedded in a cellulose floating matrix that is produced by AAB. Recently, high-throughput sequencing (HTS) has deeply investigated the relative abundance of the microbial community that is involved in the fermentation of green and black teas [2,3]. In particular, authors reported the dominant presence of *Komagataeibacter* spp. and *Acetobacter* spp. and less than 1% of *Lactobacillus* spp., among bacteria; *Zygosaccharomyces* spp. and *Brettanomyces* spp. are the most abundant yeasts [2]. Kombucha is known for its nutraceutical properties and it has been consumed in Asia for thousands of years, with its preparation dating back approximately to 220 B.C. [4]; in the last ten years, the market interest has moved to the whole world [5], and also home brewing is now a common practice. Substrates, other than black and green teas, can also be used, such as Jerusalem artichoke tuber extracts, wine, milk, fruit juices, and plant infusions [6], bringing nutritional and health benefits that are based on the selected cultivation medium [7,8,9]. Kombucha analysis is mainly focused on the chemical compounds which confer to the fermented beverage its nutritional and antioxidant value. In particular, at the end of the fermentation process (10–20 days) at a temperature in the range of 20–30 °C, Kombucha is rich in organic acids (acetic, glucuronic, gluconic acids), vitamins and tea polyphenols, and the low pH avoid bacterial contamination [6]. The beverage has a refreshing taste and consumers have the feeling that Kombucha is beneficial and improve digestion with a regular consumption [10]. Green and black tea have received considerable attention in recent years as functional beverages due to the high amount of functional compounds, such as polyphenols, flavonoids, and saponins [11]; polyphenols have been widely studied also in kombucha and they basically increase along the fermentation process [12,13,14]. The fermentation length, temperature, tea quality, sucrose concentration, and the fermenting microbial consortia strictly influence the chemical and anti-oxidant properties of the final beverage [6,10]. In particular, tea quality and composition varies with the species, season, age of the leaves (plucking position), climate, and horticultural practices [15,16], thus immediately influencing the polyphenols composition of teas and consequently of the fermented product. Moreover, the positive evidence in human health is difficult to show because of the difficulty to organize randomised and controlled intervention trials, which are also very expensive. However, several authors reported a wide spectrum of beneficial activities, studies in vitro or in animal models, including antimicrobial activity against foodborne and human pathogens, hepatic detoxification in rats, anti-inflammatory, hypocholesterolemic, anti-proliferative, and hypoglycemic activities [6,17]. To date, only one study has been applied in human subjects research, examining the health benefits of kombucha [18].

In this work, kombucha was prepared from a consortium of symbiotic microorganisms using two traditional substrates, green and black teas, and a never tested substrate, the tea obtained from rooibos (*Aspalathus linearis*) leaves. Microbial diversity was analysed during fermentation both in the cellulolytic pellicles and in the corresponding kombuchas, using culture-dependent methods and Illumina high-throughput sequencing. Additionally, total polyphenols, flavonoids, and anti-oxidant activities were monitored and substrate and metabolite concentrations were quantified. We also investigated whether the kombucha from rooibos exerts a possible protection/restoring effect in mouse fibroblast cell lines against the oxidative stress induced by H_2_O_2_. This work provides a deep insight on traditionally prepared Kombuchas and gains new knowledge on the use of rooibos herbal tea, which has never been considered as a substrate, in spite of its well-known bioactivity and potential health benefits [19,20]. 

## 2. Materials and Methods 

### 2.1. Kombucha Fermentation: Batches Preparation

The consortium of microorganisms, consisting of a cellulose biofilm, was purchased from Happy Kombucha (Eastbourne, Great Britain) and was shipped refrigerated. 30 g of pellicle was inoculated with its liquid (100 mL) in green tea, previously prepared with 1 L of water and sucrose (80 g/L), by adding 8 g/L of dried leaves for 3 min with water at 74 °C. The fermentation was carried out for 14 days at 27 ± 1 °C; the obtained fresh kombucha biofilm and 100 mL of the liquid was used as inoculum for the following infusion teas: green tea “Sencha”, 8 g/L of dried leaves at 74 °C for 3 min; black tea “Ceylon”, 8 g/L of dried leaves at 95 °C for 3 min; and, rooibos (*Aspalathus linearis*), 8 g/L of dried leaves at 95 °C for 10 min [21]. All of the glass beakers and cotton caps for the preparation of the fermentation batches were sterilized at 121 °C for 20 min. For each tea, three independent fermentation batches were prepared and incubated at 27 ± 1 °C for 14 days; samples (biofilm and liquid) were collected after 7 and 14 days of fermentation for chemicals and microbiological analysis. Samples were named, as follows: 

(1) Biofilm (F) was collected after seven days of fermentation: (a) F_7_B (B = black tea), (b) F_7_G (G = green tea), (c) F_7_R (R = rooibos tea); (2) Kombucha (K) collected after seven days of fermentation: (a) K_7_B (B = black tea), (b) K_7_G (B = black tea), and (c) K_7_R (R = rooibos tea); (3) Biofilm collected after 14 days of fermentation: (a) F_14_B (B = black tea), (b) F_14_G (G = green tea), (c) F_14_R (R = rooibos tea); and,(4) Kombucha collected after 14 days of fermentation: (a) K_14_B (B = black tea); K_14_G (G = green tea); K_14_R (R = rooibos tea).

### 2.2. Culture-Based Microbiological Investigations 

For LAB and yeast enumeration, serial dilutions were prepared starting from 1 mL of liquid and 10 g of biofilm at day 14. The biofilm was previously homogenized in 90 mL of buffered peptone water (VWR, Milano, Italy) with a stomacher (Stomacher 400 circulator, Seward Ltd, Technology Centre, Worthing, West Sussex, UK). Dilutions from 10^−2^ to 10^−8^ were plated on de Man Rogosa Sharpe (MRS) agar (Biolife, Milan, Italy) with 0.1% cycloheximide (Sigma-Aldrich, Milan, Italy) and Sabouraud Dextrose Agar (SA; Biolife, Milan, Italy) containing 100 mg/L chloramphenicol (Sigma-Aldrich, Milan, Italy) for LAB and yeasts, respectively. Analyses were performed in triplicate. Plates were incubated at 30 °C for 48–72 h and 25 °C for 72 h, respectively; the number of colony forming units (CFU/mL) was recorded and counts made for each isolation medium. Since AAB are known to be extremely difficult to cultivate in laboratory media [22], the enrichment culture approach was preferred according to Yamada et al. [23] with slight modifications. 10 mL of kombucha was mixed with 30 mL of enrichment medium containing 0.5% glucose, 0.3% yeast extract, 0.05% KH_2_PO_4_, 0.05% NaH_2_PO_4_, 0.005% MgSO_4_, 2.0% ethanol, 1.0% acetic acid 1% and 1.0% cycloheximide. When microbial growth occurred, the microorganisms were streaked on WL agar (Biolife Italiana S.r.l., Milano, Italy). From SA and WL agar, isolated colonies (100–200) were re-streaked and purified. For long-term storage, purified isolates were stored at −80 °C with their respective liquid medium containing 20% glycerol.

### 2.3. Grouping and Molecular Identification of AAB and Yeast Isolates

Grouping of AAB isolates was performed by Random Amplification of Polymorphic DNA (RAPD)-PCR, as described by Di Gioia et al. [24], after DNA extraction with the Wizard^®^ Genomic DNA Purification Kit (Promega, WI, USA). Cluster analysis of the RAPD-PCR profiles was carried out using Bionumerics 7.1 (Applied Maths, Sint-Martens-Latem, Belgium) using Dice’s Coefficient of similarity with the un-weighted pair group method arithmetic averages clustering algorithm (UPGMA). Based on the genotypic grouping, representative isolates were selected and the 16S rRNA gene amplified and sequenced according to Gaggìa et al. [25]. Yeast cells from 48 h old fresh colonies growing on SA plate were collected and suspended in 30 μL of Y-PER (Thermo Fisher Scientific, Waltham, MA, USA); suspension was vortexed for 15 s, incubated under 98 °C for 5 min, centrifuged, and the resulting supernatant diluted 1:5 before PCR analysis. Molecular identification of yeast species was carried out by analysis of the restriction digestion pattern generated from PCR amplified internal transcribed spacer region along with 5.8S rRNA gene (ITS1-5.8S-ITS2), as described by Jeyaram et al. [26] with *Rsa*I instead of *Cfo*I. Sequencing of the D1/D2 domain of the large-subunit (26S) ribosomal DNA was performed for major groups of isolates according to Jespersen et al. [27]. 

The purified and amplified products were delivered to Eurofins MWG Operon (Ebersberg, Germany) for sequencing. Sequence chromatograms were edited and analysed using the software programs Finch TV version 1.4.0 (Geospiza Inc., Seattle, WA, USA). DNAMAN software (Version 6.0, Lynnon corporation, Pointe-Claire, QC, Canada) was applied to obtain consensus sequences whose assignments to species or genera was investigated by nucleotide BLAST [28]. Finally, the sequences were reported to the GenBank tool [29].

### 2.4. Dynamics of Bacteria and Yeast Population during Fermentation

#### 2.4.1. DNA Extraction 

Total DNA extraction from kombucha samples (biofilm and liquid) collected at 7 and 14 days were carried out, using the Wizard^®^ Genomic DNA Purification Kit (Promega, WI, USA) with some adjustments, prior to protein precipitation. 1.5 g of biofilms were cut with a sterile scalpel and transferred in tubes containing 3 mL of deionized H_2_O and 200 mg/mL of cellulase (C1184, Sigma-Aldrich, Milan, Italy). Suspensions were incubated overnight at 27 ± 1 °C, homogenized, and centrifuged (10.000× *g* for 5 min). 100 mL of liquid kombuchas were centrifuged (10.000× *g* for 10 min) to obtain a pellet. The obtained pellets from the different samples were mixed with 500 µL of H_2_O and 500 µL of Y-PER (Thermo Fisher Scientific, Waltham, MA, USA) and then subjected to mechanical (by adding 0.2 g of glass beads in the MO BIO Vortex Genie^®^ 2 for 10 min) and physical (95 °C for 10 min) lysis. Finally, DNA was rehydrated with 50 µL of Rehydration Solution (Promega, Madison, WI, USA) and tubes were incubated at 65 °C for 1 h. DNA purity and yield was determined spectrophotometrically at 260 and 280 nm (Infinite^®^ 200 PRO NanoQuant, Tecan, Mannedorf, Switzerland) and then stored at −20 °C.

#### 2.4.2. Library Preparation and Sequencing

The distribution of the bacterial and eukaryotic amplicons was determined by NextSeq high-throughput (HTS) based sequencing of the partial 16S rRNA gene and internal transcribed spacer two (ITS2) gene amplicon HTS, according to Krych et al. [30] and Haastrupt et al. [31], respectively. The amplified fragments with adapters and tags were purified and normalized using custom made beads, pooled, and subjected to 150 bp pair-ended NextSeq (V3 region 16S rRNA) and 250 bp pair-ended MiSeq (Internal Transcribe Spacer 2, ITS2) sequencing. The raw dataset containing pair-ended reads with corresponding quality scores were merged and trimmed using the following settings, -fastq_minovlen 100, -fastq_maxee 2.0, -fastq_truncal 4, and -fastq_minlen of 130 bp for V3 region 16S rRNA, and 16bp for ITS2 amplicons. De-replicating, purging from chimeric reads, and constructing de-novo zero-radius Operational Taxonomic Units (zOTU) were conducted using the UNOISE pipeline [32] and taxonomically assigned with –sintax [33] coupled to the EZtaxon [34] for 16S rRNA gene and UNITE [35] for ITS2 as references.

#### 2.4.3. Bioinformatic Analysis

Low persistent and low abundant OTU’s were discarded to avoid noise. OTU’s, which persisted in less than 5% of the samples, were discarded, however still maintained an average total abundance that was close to 98%. Cumulative sum scaling (CSS) [36] was applied for the analysis of beta-diversity to counteract that a few OTU’s represented a majority of count values, and since CSS have been benchmarked with a high accuracy for the applied metrics [37]. CSS normalisation was executed with Quantitative Insight into Microbial Ecology 1.9.13 (QIIME 1.9.1) normalize_table.py. [38]. Alpha-diversity analysis was based on raw read counts to avoid bias with rarefaction [39]. QIIME 2 (2018.4 build 1525276946) plugins were used for subsequent analysis steps of alpha- and beta diversity statistics [38]. Beta-diversity analysis was represented by Bray Curtis dissimilarity. Wilcoxon Rank Sum Test evaluated pairwise taxonomic differences, whereas ANOSIM and Kruskal Wallis was used to evaluate multiple group comparisons.

### 2.5. Chemical Analysis 

#### 2.5.1. Sugar and Acid Organics Analysis

Two samples of kombucha at 7 and 14 days of fermentation, for each substrate and batch, was filtered through 0.22 μm nylon filters (Frisenette, Knebel, Denmark) and used for sucrose, fructose, glucose, glucuronic acid (GlcUA), acetic acid (AA), and ethanol (EToH) quantification by High Performance Liquid Chromatography (HPLC), as described by [40]. Briefly, a 1100 series HPLC connected to a 1260 Infinity II series RI detector (Agilent Technologies, Santa Clara, CA, USA) was used with a Rezex ROA-Organic Acid H+ (8%) column (Phenomenex, Torrance, CA, USA) with a flow rate of 0.5 mL/min for 30 min per sample.

#### 2.5.2. Total Polyphenols and Flavonoids

The amount of free phenolic compounds in kombucha samples were determined according to the Folin–Ciocalteu procedure [41]. 20 μL of kombucha tea was mixed with 0.1 mL of Folin–Ciocalteu reagent and 2 mL of purified water, and, after 5 min of incubation, 1 mL of 15% Na_2_CO_3_ was added. The mixture was measured at 765 nm after 2h at dark room temperature. Gallic acid was used as a standard and the concentration was expressed as micromoles of gallic acid equivalents (GAE) per g of dry weight (DW). For flavonoid measurements, according to Adom et al. [42], appropriate dilutions of kombucha tea were mixed with sodium nitrite, followed by a reaction with aluminium chloride. Solution absorbance at 510 nm was immediately measured and the flavonoid content was expressed as micromoles of catechin equivalent (CE) per g DW. 

#### 2.5.3. Antioxidant Activity

The total antioxidant activity of kombuchas was measured by ferric ion reducing power (FRAP) assay and 2,2-diphenyl-1-picrylhydrazyl radical (DPPH) assay with some modifications, according to Benzie et al. [43] and Floegel et al. [44], respectively. FRAP assay was performed from 10 μL of kombucha mixed with 70 μL of FRAP working solution, prepared mixing a 10:1:1 solution of 0.3 M acetate buffer, 10 mM TPTZ solution in 40 mM HCl, and 20 mM ferric chloride. After 60 min at dark conditions, the absorbance at 593 nm was measured. FRAP antioxidant activity was expressed in mmoL Fe^++^/g of dry weight (DW) and values were compared with a standard curve of Ferrous sulfate. For DPPH assay, 12.5 μL of each sample and 37.5 μL H_2_O were mixed with 2.95 mL of DPPH 100 μM. After 30 min at dark room temperature, the decrease in absorbance at 517 nm was measured. DPPH antioxidant activity was expressed as mmol Trolox/g DW.

#### 2.5.4. Catechins Identification and Quantification by HPLC-MS/MS

Standard solutions were prepared by dissolving pure compounds of (+)-catechin (C), (−)-epicatechin (EC), (+)-gallocatechin (GC), (−)-epigallocatechin (EGC), (−)-catechingallate (CG), (−)-epicatechingallate (ECG), (−)-gallocatechingallate (GCG), and (−)-epigallocatechingallate (EGCG) (Sigma-Aldrich, Milan, Italy) at a concentration of 1.0 mg/mL as reported in Naldi et al. [45]. Kombucha from black, green, and rooibos tea were filtered and injected onto a Waters e2695 Alliance HPLC System coupled with a Waters ACQUITY QDa Mass Detector. A Supelco Analytical C18 colomn (Sigma-Aldrich, Milan, Italy) (15 cm × 4.6 mm, 5 μm) was selected. The mobile phase consisted of (A) water + 0.1% formic acid and (B) ACN + 0.1% formic acid. A gradient experiment was performed from 12 to 21% B in 15.0 min, from 21 to 25% B in 3.0 min and from 25 to 100% B in 3.0 min. Solvent B returns to 12% in 0.3 min, and maintained for 3.7 min to re-equilibrate the column. The flow rate, the injection volume, and column temperature were 1.0 mL min^−1^, 15 μL, and 30 °C, respectively. The ionization source was used in the ESI negative mode, using single ion recording (SIR). Optimal cone voltage was set at 20 V and the capillary voltage at 0.8 kV; the source temperature was maintained at 120 °C; and, the desolvatation gas temperature at 600 °C. Data acquisition, data handling, and instrument control were performed using the Empower 3 software.

### 2.6. Cell Cultures, Oxidative Cell Treatments and MTT Assay

L929 mouse fibroblasts (ATCC-CCL1) were cultured with DMEM (Gibco) added with 10%, fetal bovine serum (Gibco), 1 mM L-glutammine (Gibco), and 1% penicillin-streptomicyn (Gibco). Cells were treated for 24 h with rooibos kombuchas fermented for 7 (K_7_R) and 14 days (K_14_R). Before the cell treatment, all of the kombuchas were diluted with DMEM at the same concentration (50 μg gallic acid equivalents (GAE)/mL). H_2_O_2_ and water were used as negative and positive control, respectively. For oxidative stress assays, cultured fibroblast cell viability was evaluated after treating cells with 75 μM H_2_O_2_ in PBS for 20 min, according to the procedure that was proposed by Leoncini et al. [46]. Data are expressed as percentage of viable cells with respect to untreated controls (water).

L929 fibroblasts were plated in 96-well tissue culture plate (8000 cells/well). After treatments, proliferative cells were detected by 3-(4,5-dimetiltiazol-2-il)-2,5-difeniltetrazolio (MTT) assay, according to the ISO 10993-5 International Standard procedure [47]. The main purpose of the ISO 10993-5 procedure is to define a scheme for testing the *in vitro* cytotoxicity of different extracts according to a multi-step approach. Briefly, cells were incubated with MTT solution (1 mg/mL, Life Technologies, Carlsbad, CA, USA) at 37 °C for 2 h. Subsequently, cells were solubilized with isopropanol and the formazan dye formation was evaluated by scanning multi-well spectrophotometer at 540 nm. The results were expressed as viability percentage with respect to untreated control (water). 

### 2.7. Statistical Analysis

The quantification of chemical compounds was performed on triplicate samples of each kombucha accession. Data of chemical analyses were represented as mean ± standard deviation. The cell tests were carried out with six replicates for each treatment and data were expressed as mean values of three different experiments. Statistical analysis was performed with R software [48]. Normal and homoscedastic data were analyzed with ANOVA and Tukey post-hoc tests with Bonferroni correction. Non-normal homoscedastic data were analyzed with the nonparametric Kruskall–Wallis test and Dunn’s post-hoc test with Bonferroni correction. Differences were considered to be significant at a *p* value < 0.05.

## 3. Results

### 3.1. Culture-Based Enumeration and AAB and Yeast Isolates Identification 

Results on Lactic Acid Bacteria (LAB) and yeasts enumeration from biofilms and kombuchas are listed in Table S1. No growth was observed on the MRS agar plate. Yeast count ranged from 6.83 ± 0.02 to 7.97 ± 0.08 log_10_ CFU/mL, with the lowest values found in F_14_B and K_14_B; the F_14_G yeast count was significantly higher when compared to the other films and kombuchas. 120 AAB isolates were obtained after the enrichment step and re-streaked for RAPD-PCR analysis. Clustering analysis divided the profiles into 11 sub-clusters (Figure S1); similarities within each cluster was nearly or higher than 90%. Strains isolated in the biofilms and kombuchas of the three teas were scattered among the sub-clusters. However, isolates from F_14_R, G_14_K, and R_14_K generally belonged to the same sub-cluster. Representative isolates from each sub-cluster were then identified by 16S rDNA sequencing. The identification and the taxonomy assignment are shown in Table 1. High similarities (99–100%) were found among the isolates that were obtained during the present study. Most of the AAB were mainly ascribed to *Komagataeibacter* spp. and *Gluconobacter* spp. In some cases, the identification reached the species level with different abundance among the substrates (Table S2); *Komagataeibacter intermedius* was particularly abundant in films and kombucha from black and green teas, while *Gluconobacter entanii* was detected almost exclusively in kombucha from rooibos. *Komagataeibacter rhaeticus* was isolated in all substrates, but preferentially in F_14_G and K_14_G.

Yeasts (130 isolates) were grouped based on the restriction analyses profiles, which divided the isolates in two groups (Figure S2). Identification of representative selected isolated (Table 2) showed the presence of two species: *Brettanomyces bruxellensis* and *Zygosaccharomyces parabailli*. *B. bruxellensis* was particularly abundant in all kombuchas and in F_14_G and F_14_B; whereas, *Z. parabailli* was dominant in F_14_R (Figure S2).

### 3.2. High-Throughput Sequencing of 16S rRNA Gene and ITS Region Amplicons

**Figure 1:**
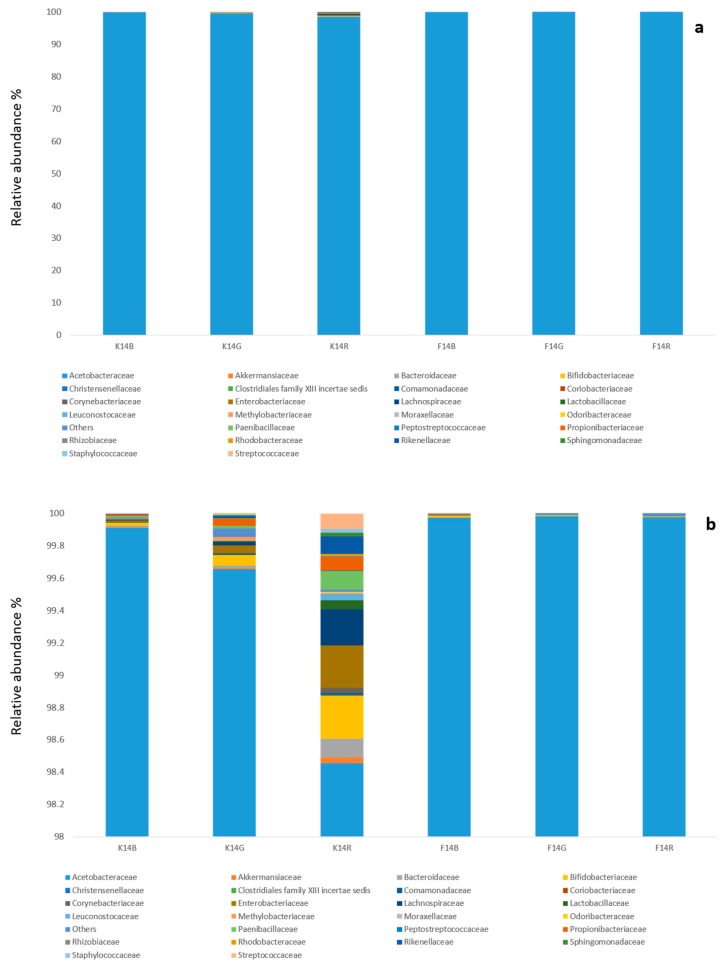
The influence of substrate and matrix on bacterial community structure of kombucha (family level), after 14 days of fermentation, as determined by 16S rRNA gene amplicon sequencing, showing in (**a**) the relative abundance from 0 to 100% and (**b**) the focus in the range between 98% and 100%.

**Figure 2:**
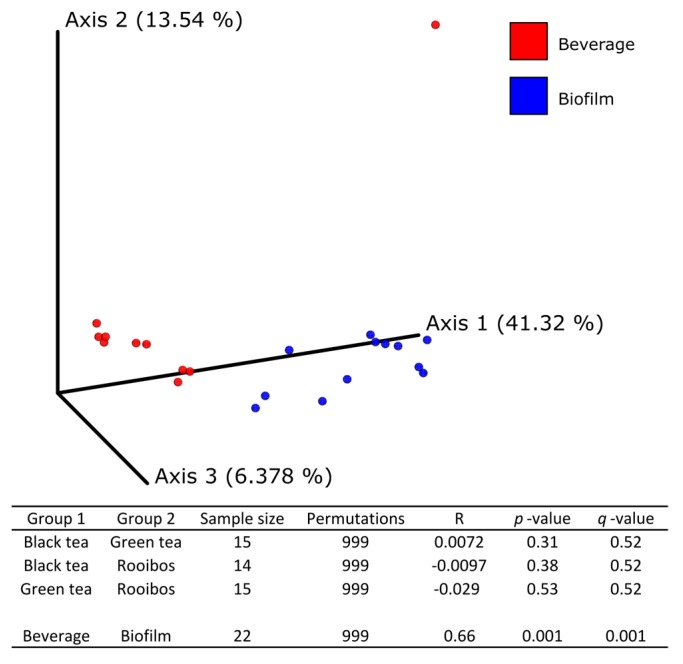
The influence of matrix (plot) and substrate (only table) on kombucha bacterial community structure after 14 days of fermentation illustrated as a PCoA plot. Bray–Curtis dissimilarity index determined by 16S rRNA gene amplicon sequencing (pairwise ANOSIM, 999 permutations).

Following high throughput sequencing of 16S rRNA gene, 108,351–271,740 reads per sample with a median of 217,303 paired end reads were obtained from F_14_ and K_14_ samples from all of the substrates after cleaning and quality control check. Five bacterial phyla were identified through HTS. Most OTUs were assigned taxonomically at the genus or species level. The bacterial community composition (Figure 1, Table S3) was dominated by Proteobacteria, in particular, the family Acetobacteraceae, which accounted for >99% and for >97% of reads in the biofilm and kombucha, respectively, for all tea substrates. Among Proteobacteria, Enterobacteriaceae were found in K_14_R lower than 0.3%. Members of Acetobacteraceae consisted only of the genera *Komagataeibacter* spp. with *Komagataeibacter saccharivorans* the only detected species. Among the Firmicutes, different families (Lactobacillaceae, Paenibacillaceae, Staphylococcaceae, Streptococcacea, Lachnospiraceae, etc.) were detected; in K_14_R, they reached a relative abundance lower than 0.3%. In K_14_R Bacteroidetes (family Bacteroidaceae) and Actinobacteria (family Bifidobacteriaceae) accounted for 0.11% and 0.26%, respectively. Bacterial alpha diversity was significantly different between kombucha and biofilm at 14 days of fermentation (*q*-value = 0.000124), while there was no significant difference in alpha diversity among tea types (Figure S3a–c). Beta diversity of the bacterial community was significantly separated between kombucha and biofilm (*q*-value = 0.001), but not among tea type, as judged by the Bray–Curtis dissimilarity index (Figure 2a,b).

**Figure 3:**
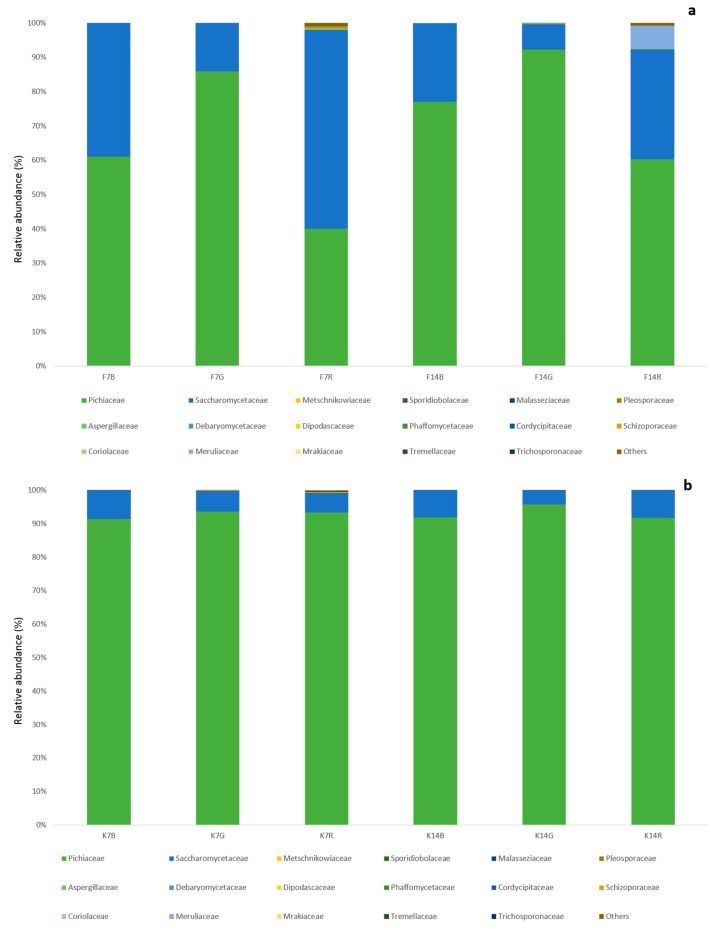
The influence of substrate and matrix on bacterial community structure of kombucha (family level) after 7 (**a**) and 14 (**b**) days of fermentation, as determined by internal transcribed spacer two (ITS2) gene amplicon sequencing.

High throughput sequencing of ITS region 2 amplicons obtained 44,795–140,438 paired end reads per samples, with a median of 105,310 after cleaning and quality control check. The fungal community of both biofilm and kombucha (Figure 3, Tables S4 and S5) was dominated by the phylum Ascomycota, in particular, by the family Pichiaceae (>60% and >91%, respectively), with an unidentified Pichiaciae *Dekkera* spp., as the main abundant genera. 

**Figure 4:**
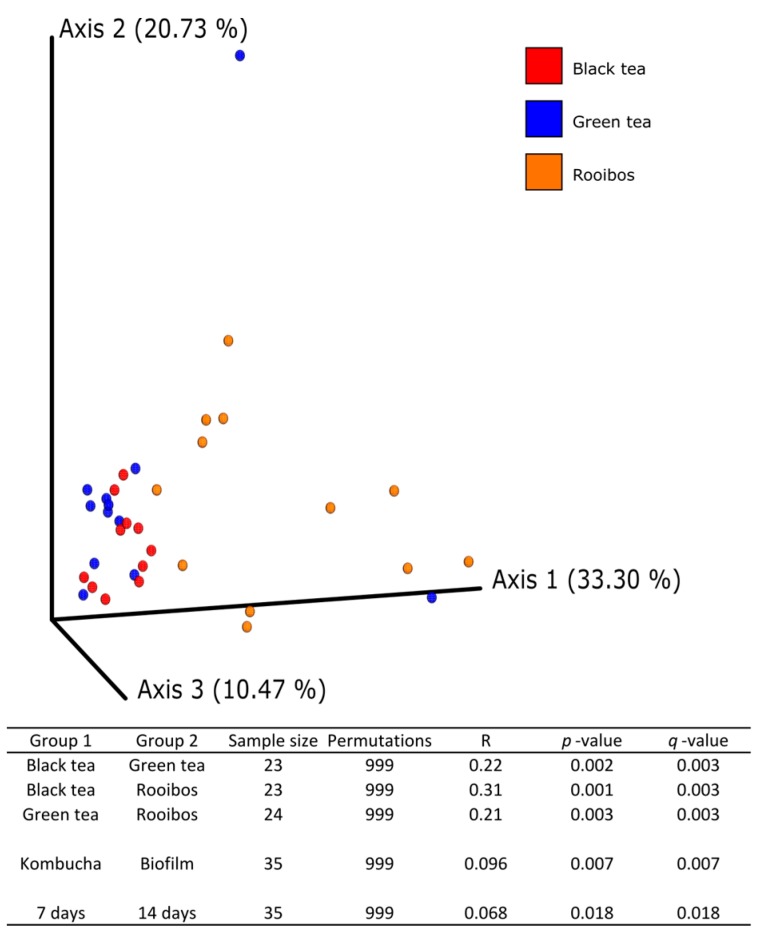
The influence of substrate (plot, table), matrix (only table) and fermentation time (only on kombucha fungal community structure after 14 days of fermentation illustrated as a PCoA plot of Bray-Curtis dissimilarity index determined by ITS2 gene amplicon sequencing (pairwise ANOSIM, 999 permutations).

The second most abundant family was Saccharomycetaceae that in all kombucha samples was lower than 8.5%, but in the biofilm the abundance increased, reaching its maximum in F_7_R (57.9%) and the ratio between the two families was inverted. Deeper analysis of ITS sequence allowed, at the species level, to assign some members of the Pichiaceae family to *Brettanomyces bruxellensis* with a low abundance (0.4–2.0%). Among Saccharomycetaceae, the genus *Zygosaccharomyces* was dominant with *Zygosaccharomyces parabailli,* reaching a relative abundance of 3–35%. Fungal alpha diversity as determined by the Shannon index showed a significant difference between kombuchas and biofilms (*q*-value 0.000028), but no significant difference between tea type was observed, nor at 7 nor after 14 days of fermentation (Figure S4a–c). Alpha diversity comparison for 7 and 14 days of fermentation showed that the difference between Kombucha and biofilm was greatest at 7 days of fermentation (*q* = 0.00053 vs. *q* = 0.019), but still no significant difference among substrates was detected. On the other hand, after 14 days, green tea was significantly different from black tea (*q* = 0.049) and green tea and rooibos were near significantly different (*q* = 0.056). No difference was observed between black and rooibos kombuchas (*q* > 0.05) (Table S6). Comparisons of Fungal Bray-Curtis beta diversity showed that the yeast community composition was significantly different among all three tea substrates (*q* = 0.003), as well as between 7 and 14 days of fermentation (*q* = 0.018) and between biofilm and kombucha (*q* = 0.007) (Figure 4). As observed for alpha diversity comparisons, samples showed greater difference between biofilm and kombucha at 7 days, whereas the effect of the substrate was reater at 14 days (Table S7). 

### 3.3. Chemical Analysis 

#### 3.3.1. Sugars and Acid Organics

Sugar consumption and organic acid production in the three kombuchas at 7 and 14 days of fermentation are shown in Table 3 and Table 4. The total sucrose generally decreased in all kombuchas along time with the lowest concentration found in K_14_B and in K_14_G (26.13 ± 0.43 and 6.21 ± 0.14 mg/mL). Glucose and fructose concentration showed an increase during the fermentation process, which is significant only in K_14_R with respect to K_7_R. Organic acids (GLcUA and AA) evidenced a significant increase in all kombucha during the fermentation, whereas there is no significant difference among the substrates. GlcUA significantly increased only in K_14_B compared to K_7_B (3.22 ± 0.63 vs. 1.36 ± 0.08 mg/mL); KR had an increase, although not significant, close to the double, and KG evidenced a slight increase. AA significantly increased at day 14 in all kombuchas with the highest amount produced in K_14_B (9.18 ± 0.15 mg/mL). Ethanol production increased along the fermentation but not significantly; both at day 7 and 14, ethanol quantification in KB and KR was significantly different (4.69 ± 0.05 vs. 0.64 ± 0.01 mg/mL and 5.83 ± 0.08 vs. 1.14 ± 0.01 mg/mL) but not for KG. 

#### 3.3.2. Polyphenols, Flavonoids and Antioxidant Activity 

The analysis of polyphenol and flavonoid concentration showed a different trend during the fermentation process (Table 5). KG had a significant increase of the polyphenol and flavonoid content during fermentation, with a maximum peak at day 7, whereas K_14_G showed a significant decrease (−33% and −18%, respectively). K_0_B samples had the highest values of polyphenols and flavonoids, significantly different from K_7_G and K_14_G, but during fermentation, the compounds were subjected to a decrease by 18% and 22% respectively, without showing significant differences to K_7_G, and K_14_G. KR showed polyphenol concentrations that were significantly lower than KG and KB, with little differences in polyphenol content during the fermentation process (−9.8%). The flavonoid content in all kombucha statistically decreased along the fermentation (except for K_7_B and K_14_B), and KR showed the highest flavonoid concentration. The decrease was similar to KG and KB (−20%). The antioxidant activity was monitored on the three substrates during the fermentation process with two common assays, DPPH and FRAP (Table 5). Both methods evidenced that K_0_G, K_0_B, and K_0_R have the lowest values of antioxidant activities, while K_7_G, K_7_B, and K_7_R has the highest ones. Moreover, K_7_G has the highest antioxidant activities, which were statistically significant when compared to all kombuchas at 7 and 14 days of fermentation. 

#### 3.3.3. Catechins Identification and Quantification by HPLC-MS/MS

The method linearity was estimated by plotting the obtained peak area of each analyte versus the corresponding analyte concentration expressed as µg/mL. Table 6 showed the calibration parameters as regression equations and correlation coefficients (R^2^) for analyses of the standard mixture. As can be observed, good linearity was obtained over the whole concentration range for all analytes, with R^2^ values that were systematically higher than 0.9817. 

The coupling of HPLC with MS detection, without any sample preparation, was evaluated for the determination of catechins. For the HPLC–MS experiments, a 25-min method was selected, as it presents the best compromise between analysis time and resolving power. The negative mode and the single ion recording (SIR) were selected to attain the better intensity and sensitivity. Epi form of catechins (EC, EGC, ECG, and EGCG) with the same m/z value of non epi-form (C, GC, CG, and GCG) were identified using the retention time of standard samples.

The estimated amounts of catechins, expressed as mg/g of tea leaves, were reported in Table 7. GCG and CG were not found in any teas and kombuchas; in addition, no catechins were detected in KR. Overall, KG tea showed an extremely higher catechins content when compared to KB tea. Before fermentation, EGC and EGCG resulted to be the most abundant catechins, respectively, in KG and KB. KB and KG statistically decreased their content during the fermentation with the reduction amount being higher in KG than KB. However, during the fermentation, EGC was higher in K_14_G than in K_0_G, and EC and GC increased from day 7 to day 14.

#### 3.2.4. Effect of Rooibois Kombuchas on Fibroblasts Proliferation

**Figure 5:**
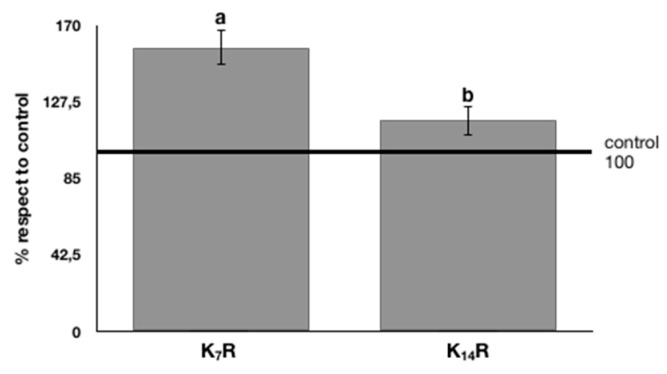
Effects of rooibos kombuchas on L929 cell proliferation; data, expressed as % of control, represent mean ±2 SEM of three independent experiments. Statistical analysis of differences was carried out by two-way ANOVA followed by Fisher’s LSD as post-hoc test. Different letters represent statistical significance (*p*  ≤  0.05).

In order to study the effect of the rooibos kombuchas on cell proliferation, mouse fibroblasts L929 were treated for 24 h with kombuchas diluted at the same concentration (50 μg GAE equivalents/mL). Cell viability (Figure 5) showed that rooibos kombucha after seven days of fermentation induced a significant upregulation of cell proliferation, with a mean increase of cell viability equal to +58% with respect to the untreated control (water). In contrast, the K_14_R induced a significant positive effect on cell proliferation, but to a lesser extent with respect to K_7_R.

**Figure 6:**
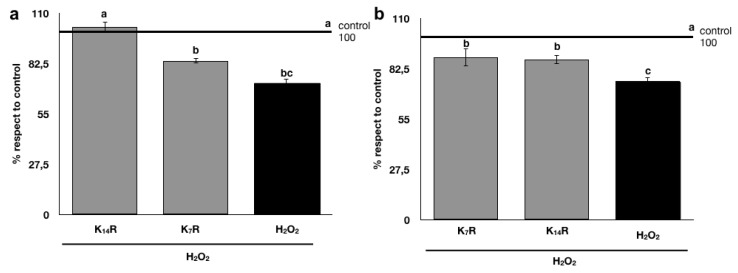
L929 cell proliferation after oxidative stress: (**a**) “Curative model” and (**b**) “Preventive model”. Data, expressed as % of control, represent mean ±2 SEM of three independent experiments. Statistical analysis of differences was carried out by two-way ANOVA followed by Fisher’s LSD as post-hoc test. Different letters represent statistical significance: (*p*  ≤  0.05).

In order to mimic a state of oxidative stress, mouse fibroblasts were treated with an oxidizing agent, 75 μM H_2_O_2_ for 20 min, and cell proliferation was measured after the treatments by MTT assay. As shown in Figure 6, the H_2_O_2_ application significantly reduced the cell proliferation when compared to the untreated control (water). The tested kombuchas exerted different effects as a function of the fermentation time duration (7 or 14 days). In particular, in the “curative model” (i.e., H_2_O_2_ oxidative stress applied to fibroblasts before the treatment with kombucha) (Figure 6a), K_14_R completely restored cell viability, which resulted in being comparable to the untreated control, while K_7_R did not restore cell proliferation in a significant way. In the “preventive model” (i.e., H_2_O_2_ oxidative stress that was applied to fibroblasts after the treatment with kombucha) (Figure 6b), both K_7_R and K_14_R were able to partially restoring cell viability with respect to the negative control (H_2_O_2_ treatment), even if none of them were able to completely protect the cell towards the toxicity effects induced by H_2_O_2_ to the negative control (H_2_O_2_ treatment).

## 4. Discussion

Fermented beverages are more and more consumed as consumers see the fermentation as a gentle method of preservation, improving well-being and reducing the risk of disease. Consumers of kombucha are increasing worldwide, the brew being a delicious combination of a delicate sour taste, antioxidant properties of tea extracts, and the potentially beneficial effect of fermenting bacteria. Kombucha prepared from black and green teas have been widely studied, although in depth microbiological studies on both bacteria and yeasts with a combination of culture dependent and molecular techniques have not been performed up to now. On the contrary, to the best of our knowledge, kombucha prepared from rooibos has not been studied yet. Rooibos tea was selected as a substrate, because of its reported beneficial action against human diseases [49,50], thus hypothesizing a possible additional nutraceutical value beyond the fermentation process. In addition to the focus on a new substrate, this study examines the influence of the different tea substrates on the microbial composition and the presence of antioxidant molecules. 

The combination of culture-dependent and culture-independent techniques is a useful approach to mitigating the main problems that arise from culturing (e.g., low-throughput, sensitivity of selective media, slow growing microorganisms, temperature sensitivity); in addition, it allows a deeper insight into microbial diversity and the isolation of some strains of biotechnological interest. Both culturing and HTS showed the dominance of *Komagataeibacter* spp. in all biofilms and kombuchas, and HTS conferred this abundance to *K. saccharivorans* with no differences among tea types. This is in accordance with other published studies [3], which mainly found the same species in green and black Kombucha fermented at 30 °C, a temperature that is comparable to that used in this study. Overall, most of the authors performing culture-independent analysis are in substantial agreement with these findings at the genus level [2,51], probably determined by a selection process due to the harsh acidic environment at the end of the fermentation process (pH 2.8, as detected in our kombuchas). However, differences can be detected at the species level, based on the assumption that the starter cultures and fermentation temperature may confer the microbiological connotation to the final beverage [2,3].

Culture-dependent techniques allowed for the isolation of a limited number of AAB identified as *K. intermedius* and *K. rhaeticus*, which have been previously identified in fruit juices [52]. These species have notably high ethanol and acetic acid tolerance, they are GlcUA producers, and they produce cellulose at a high rate, [53,54]. The species *K. intermedius* has been used in a wide range of food, industrial, and pharmaceutical applications, since not only it has a high ethanol and acetic acid tolerance but it is also an effective producer of exopolysaccharides, nanofibrillated cellulose, and GlcUA [55]. Indeed, it was mainly isolated from KB and KR, which possess the highest GlcUA concentrations. *Gluconobacter entanii*, mainly isolated from KR, is a species originally found in a submerged spirit vinegar fermentation factory [56], as this species needs high acetic acid concentration and does not produce cellulose. However, due to the enrichment method, it was not possible to get a reliable percentage of their presence in the three substrates. Nevertheless, when considering the decrease in sucrose concentration and the significant increase of acetic acid during the fermentation in all substrates and the consequent GlcUA production, the AAB populations seemed to be consolidated in the beverages without any competition. Although the relative abundance of Firmicutes and Actinobacteria was low (less than 0.3%), a small community belonging to these phyla was detected, as in Marsh et al. [2] and De Filippis et al. [3]; members of both phyla were not detectable in the biofilm, but only in the fermented beverage, in particular in KR, where the lowest AA and EtOH concentrations were measured.

The yeast population that was detected in the different studies in the literature is heterogeneous [2,51], indicating different dominant genera in the different studies. Differently from Marsh et al. [2], the relative abundance of yeasts in the fermented teas did not match that of the biofilm, thereby suggesting that the fungal composition of the cellulosic pellicle used to inoculate the teas is subjected to a selection process during the fermentation, depending on the substrate as well as physical and chemical changes in the medium. Moreover, it appears that the choice of tea substrate has an increasing effect on fungal alpha diversity of kombucha as the fermentation time increases, while the difference in fungal community diversity between kombucha and biofilm decreases.

The two dominant genera found in this study are typical of kombucha [2,53,57]; in particular, *Brettanomyces* (the anamorph of *Dekkera*) has adapted to harsh and limiting environmental conditions, with low pH values [58,59], and it is also often associated with high-ethanol biotechnological habitats [60,61]. Interestingly, Nguyen et al. [53] selected a co-culture of a *B. bruxellensis* strain (KN89) and a *K. intermedius* strain (KN89) for optimal GlcUA production. Although the relative abundance of *B. bruxellensis* in our experiments is low, isolation by plating allowed for the identification of only this species within the *Brettanomyces* genus, thus hypothesizing that the unidentified *Dekkera* spp. by HTS could be ascribed to the same species. 

Chemical analysis showed that the concentrations of organic acids and monosaccharides increased during the fermentation, as already shown by other studies [12,13,62]. Nevertheless, as reviewed by Jayabalan et al. [6], chemicals are strictly correlated with many parameters (temperature, fermentation periods, pellicle origin, etc.). The different detected concentrations may be correlated with the abundance of acid producing strains as already discussed above. The highest acetic acid and GlcUA concentrations were found in KB as indicated in other published studies [13,62]. Concerning KR, any reference value is available, being the rooibos kombucha analysed for the first time in the present work; however, the increasing concentration of GlcUA with time and the low concentration of acetic acid could be considered interesting properties due to the detoxifying properties of GlcUA [63,64], and the more delicate taste that was conferred by the lower acetic acid amount. Moreover, GlcUA increases the polyphenols bioavailability [65]. With respect to antioxidant activity and cathechins, the results are in accordance with authors, stating that cathechin compounds are degraded by bacterial and yeast activity in simpler molecules, thus increasing the antioxidant power [13,66,67]. This aspect is extremely important in a fermented beverage, since antioxidant molecules of teas and plant extracts have scavenger action against free radicals, thus possibly protecting against oxidative damage. Indeed, several studies have already investigated the biological activities of Kombucha prepared from traditional substrates in in vitro cell models and animal models [20,68,69,70]. Contrary to KB and KG, KR does not contain catechins, since rooibos is not a source of cathechins and this may be the cause of the lower antioxidant activity shown by the DPPH and FRAP assays. However, it contains other molecules, including aspalathin, isoorientin, orientin, and rutin (mainly), and, at lower concentrations, iso-vitexin, vitexin, isoquercitrin and hyperoxide, quercetin, luteolin, and chrysoeryol, all with antioxidant activity [71]. It has been shown that the aspalathin and nothofagin could be potential therapeutic agents for the treatment of various severe vascular inflammatory diseases via the inhibition of the HMGB1 signaling pathway [72]. Sanderson et al. [73] showed that hot water-soluble solids from rooibos inhibited adipogenesis and affected adipocyte metabolism, suggesting its potential role in preventing obesity. These observations, therefore, supported the choice of a deeper investigation on the health benefit of rooibos fermented beverage, although the measured antioxidant activity could not be attractive. To the best of our knowledge, this is the first study investigating the possible beneficial activity of KR against the oxidative stress using murine cell models. In the present paper, significantly positive effects on limiting the oxidative stress in the adopted cell model (fibroblast, oxidation induced by H_2_O_2_ treatment before and after kombucha treatments) was observed for rooibos kombucha. The protective effect of rooibos kombucha on fibroblasts was found to be in line with Pringle et al. [74], who showed that rooibos infusion decreased cellular oxidative stress and attenuated apoptotic/necrotic cell death in human dermal fibroblasts, by using an in vitro model to mimic diabetic wounds. These results highlighted some properties that support the potential therapeutic action of rooibos and its application in wound healing.

## 5. Conclusions

Kombucha that was prepared with the three different substrates displayed a great similarity in bacterial composition and heterogeneity in the yeast community, showing, to some extent, the influence of the substrate on the fermenting microbiota. The kombucha that was prepared with rooibos, for the first time assayed in this work, has a low ethanol and acetic acid concentration, a GlcUA amount comparable to KB as well as an important effect on the recovery of oxidative damage, thus making this substrate interesting for the preparation of kombucha with health benefits. Moreover, the present study paves the way for studies aimed at the selection of new starter microorganisms that are capable of producing beneficial compounds, such as glucuronic acid.

## Figures and Tables

**Table 1:** Best-match identification of acetic acid bacteria (AAB) isolates from films and kombucha teas obtained by nBLAST.

Isolates	Source	Closest Match (% similarity *)	Accession Number
FR-3	Kombucha rooibos	*Komagataeibacter* spp. (99%)	MK099856
FR-10	Kombucha rooibos	*Komagataeibacter* spp. (99%)	MK106667
FG-14	Kombucha green tea	*Komagataeibacter* spp. (99%)	MK099857
FB-12	Film black tea	*K. intermedius* (100%)	MK099859
KB-16	Kombucha black tea	*K. intermedius* (100%)	MK099858
KG-15	Kombucha green tea	*Komagataeibacter* spp. (99%)	MK099860
KR-9	Kombucha rooibos	*K. intermedius* (100%)	MK099861
KR-17	Kombucha rooibos	*K. rhaeticus* (100%)	MK099862
KR-11	Kombucha rooibos	*G. entanii* (100%)	MK099863
KR-1	Kombucha rooibos	*Komagataeibacter* spp. (99%)	MK099864
KR-2	Kombucha rooibos	*G. entanii* (100%)	MK099865
KR-3	Kombucha rooibos	*Komagataeibacter* spp. (99%)	MK099866
KR-4	Kombucha rooibos	*G. entanii* (100%)	MK099867
KR-5	Kombucha rooibos	*G. entanii* (100%)	MK099868
KG-16	Kombucha green tea	*G. entanii* (100%)	MK099869
KG-2;	Kombucha green tea	*K. intermedius* (100%)	MK099870
KG-5	Kombucha green tea	*K. intermedius* (100%)	MK099871
KG-13	Kombucha green tea	*G. entanii* (100%)	MK099872
KB-17	Kombucha black tea	*K. intermedius* (100%)	MK099873
KG-14	Kombucha green tea	*Komagataeibacter* spp. (99%)	MK099874
KG-17	Kombucha green tea	*Komagataeibacter* spp. (99%)	MK099875
KG-18	Kombucha green tea	*Komagataeibacter* spp. (99%)	MK099876
KG-26	Kombucha green tea	*Komagataeibacter* spp. (99%)	MK099877
KG-27	Kombucha green tea	*Komagataeibacter* spp. (99%)	MK099878
FB-9	Film black tea	*Komagataeibacter* spp. (99%)	MK099879
KB-25	Kombucha black tea	*Komagataeibacter* spp. (99%)	MK099880
KR-10	Kombucha rooibos	*Komagataeibacter* spp. (99%)	MK099881
KR-16	Kombucha rooibos	*Komagataeibacter* spp. (99%)	MK099882
KR-24	Kombucha rooibos	*Komagataeibacter* spp. (99%)	MK099883

* Similarity represents the % similarity shared with the sequences in the GenBank database.

**Table 2:** Best-match identification of yeast isolates from films and kombucha teas obtained by nBLAST.

Isolates	Source	Closest Match (% similarity *)	Accession Number
YFB-2	Film black tea	*Z. parabailii* (99%)	MH930858
YFB-9	Film black tea	*B. bruxellensis* (100%)	MH930859
YFB-18	Film black tea	*B. bruxellensis* (99%)	MH930860
YFR-15	Film rooibos	*Z. parabailii* (99%)	MH930861
YFR-6	Film rooibos	*B. bruxellensis* (100%)	MH930862
YKB-1	Kombucha black tea	*Z. parabailii* (99%)	MH930863
YKB-2	Kombucha black tea	*Z. parabailii* (99%)	MH930864
YKB-9	Kombucha black tea	*B. bruxellensis* (99%)	MH930865
YKR-2	Kombucha rooibos	*B. bruxellensis* (100%)	MH930866
YKR-3	Kombucha rooibos	*B. bruxellensis* (99%)	MH930867

* Similarity represents the % similarity shared with the sequences in the GenBank database.

**Table 3:** Concentration (mg/mL) of glucose, sucrose, and fructose in Kombucha prepared with black tea (KR), green tea (KG), and rooibos tea (KR) at 7 and 14 day of fermentation.

Substrate	Glucose		Sucrose		Fructose	
	7	14	7	14	7	14
KB	11.20 ± 0.99	15.12 ± 0.64	36.23 ± 0.03	26.13 ± 0.43	4.84 ± 0.001	5.50 ± 0.13
KG	11.40 ± 0.22	15.89 ± 0.06	37.14 ± 0.09	26.21 ± 0.14	5.12 ± 0.02	6.92 ± 0.02
KR	8.60 ± 0.14 ^A^	18.10 ± 0.20 ^B^	42.08 ± 0.09	33.65 ± 0.05	4.07 ± 0.04 ^A^	8.83 ± 0.04 ^B^

(^A,B^) different letters showed significant difference between different sampling times for the same substrate (Bonferroni corrected *p* < 0.05. No letters showed no significance.

**Table 4:** Concentration (mg/mL) of glucuronic acid (GlcUA), Acetic acid and ethanol (EtOH) in Kombucha prepared with black tea (KR), green tea (KG), and rooibos tea (KR) at 7 and 14 day of fermentation.

Substrate	GlcUA		AA		EtOH	
	7	14	7	14	7	14
KB	1.36 ± 0.08 ^A^	3.23 ± 0.64 ^B^	3.18 ± 0.003 ^A^	9.18 ± 0.15 ^B^	4.69 ± 0.05 ^b^	5.83 ± 0.08 ^a^
KG	1.78 ± 0.12	1.96 ± 0.10	4.22 ± 0.02	7.65 ± 0.003	2.81 ± 0.01 ^ab^	4.18 ± 0.03 ^ab^
KR	1.70 ± 0.09	2.87 ± 0.47	1.65 ± 0.004	4.89 ± 0.02	0.64 ± 0.01 ^a^	1.14 ± 0.01 ^b^

(^a,b^) different letters showed significant difference between different substrate in the same sampling time (Bonferroni corrected *p* < 0.05); (^A,B^) different letters showed significant difference between different sampling times for the same substrate (Bonferroni corrected *p* < 0.05). No letters showed no significance.

**Table 5:** Polyphenols and flavonoids content and antioxidant activity of kombuchas 0, 7, and 14 days of fermentation.

Kombucha	Polyphenols (mg/g DW)	Flavonoids (mg/g DW)	DPPH Test (mmol TE/g DW)	FRAP Test (mmol Fe^++^/g DW)
K_0_G	74.40 ± 1.64 ^b^	16.57 ± 0.21 ^b^	0.31 ± 0.01 ^c^	0.70 ± 0.01 ^c^
K_7_G	100.33 ± 2.36 ^a^	18.49 ± 0.73 ^a^	1.31 ± 0.07 ^a^	1.75 ± 0.06 ^a^
K_14_G	67.40 ± 2.69 ^c^	15.11 ± 0.22 ^c^	0.98 ± 0.01 ^b^	1.13 ± 0.06 ^b^
K_0_B	79.38 ± 0.77 ^a^	17.97 ± 0.05 ^a^	0.31 ± 0.01 ^b^	0.68 ± 0.02 ^b^
K_7_B	64.81 ± 2.91 ^b^	14.46 ± 0.19 ^b^	0.87 ± 0.01 ^a^	0.90 ± 0.04 ^a^
K_14_B	67.20 ± 3.48 ^b^	13.87 ± 0.79 ^b^	0.85 ± 0.02 ^a^	0.86 ± 0.03 ^a^
K_0_R	43.51 ± 2.89 ^ab^	21.72 ± 0.01 ^a^	0.18 ± 0.01 ^c^	0.49 ± 0.05 ^a^
K_7_R	45.32 ± 1.36 ^a^	18.15 ± 0.52 ^b^	0.45 ± 0.03 ^a^	0.52 ± 0.01 ^a^
K_14_R	40.89 ± 1.25 ^b^	17.33 ± 0.84 ^c^	0.41 ± 0.01 ^b^	0.47 ± 0.04 ^a^

Values of each kombucha (means ± sd) with different letters are significantly different (Tukey’s test, *p*  ≤  0.05). DW: Dry Weight; TE: Trolox equivalent

**Table 6:** Calibration curves with correlation coefficient (R^2^) and m/z value for each catechins.

Analyte	Concentration range (ppm)	Calibration equation	R^2^	m/z
C_KG, KB_	0.05–2.00	y = 3.73E+06x + 1.76E+04	0.9999	290.3
EC_KG_	12.5–50	y =7.30E+05x + 1.58E+07	0.9817	290.3
EC_KB_	0.05–5	y = 3.34E+06x + 2.54E+04	0.9998	290.3
GC_KG, KB_	0.1–5	y = 3.64E+06x + 1.38E+05	0.9962	306.3
EGC_KG_	12.5–80	y = 9.09E+05x + 1.46E+07	0.9941	306.3
EGC_KB_	0.1–5	y = 2.85E+06x + 3.07E+05	0.9843	306.3
ECG_KG, KB_	0.05–1	y = 5.26E+06x − 7.05E+04	0.9999	442.4
EGCG_KG, KB_	12.5–50	y = 7.11E+05x + 5.88E+05	0.9996	458.4

C: (+)-catechin; EC: (−)-epicatechin; GC: (+)-gallocatechin; EGC: (−)-epigallocatechin; CG: (−)-catechingallate; ECG: (−)-epicatechingallate; GCG: (−)-gallocatechingallate; EGCG: (−)-epigallocatechingallate; KG: kombucha from green tea; KB: kombucha from black tea.

**Table 7:** Content of catechins in kombucha samples expressed as mg/g DW ± s.d.

Kombucha	C	EC	GC	EGC	ECG	EGCG	TOTAL
K_0_G	0.173 ^a^	2.903 ^a^	0.505 ^a^	9.086 ^b^	0.080	5.506 ^a^	18.253 ^a^
K_7_G	0.019 ^b^	1.541 ^b^	0.098 ^c^	7.084 ^c^	n.d.	1.029 ^b^	9.770 ^c^
K_14_G	0.019 ^b^	1.769 ^b^	0.110 ^bc^	9.650 ^a^	n.d.	0.296 ^c^	11.844 ^b^
K_0_B	0.083 ^a^	0.413 ^a^	0.081 ^a^	0.304 ^b^	0.095	1.209 ^a^	2.184 ^a^
K_7_B	0.015 ^b^	0.134 ^b^	0.026 ^b^	0.431 ^a^	n.d.	0.385 ^b^	0.99 ^b^
K_14_B	0.008 ^b^	0.080 ^bc^	0.003 ^b^	0.270 ^b^	n.d.	0.104 ^c^	0.464 ^c^
K_0_R	n.d.	n.d.	n.d.	n.d.	n.d.	n.d.	n.d.
K_7_R	n.d.	n.d.	n.d.	n.d.	n.d.	n.d.	n.d.
K_14_R	n.d.	n.d.	n.d.	n.d.	n.d.	n.d.	n.d.

Values of each kombucha (means ± s.d.) with different letters are significantly different (Tukey’s test, *p*  ≤  0.05). n.d.: not detected.

## Supplementary Materials

The following are available online at http://www.mdpi.com/2072-6643/11/1/1/s1, Figure S1, Figure S2, Figure S3, Figure S4, Table S1, Table S2, Table S3, Table S4, Table S5, Table S6, Table S7.
