# Supplementary material for: Kombucha Beverage from Green, Black and Rooibos Teas: A Comparative Study Looking at Microbiology, Chemistry and Antioxidant Activity
Source: Nutrients. 2018 Dec 20;11(1):1. doi: 10.3390/nu11010001 (PMC6356548; doi:10.3390/nu11010001)
Supplement: Supplementary file 1 [file nutrients-11-00001-s001.zip › Nuova cartella/Figure S2.pdf]

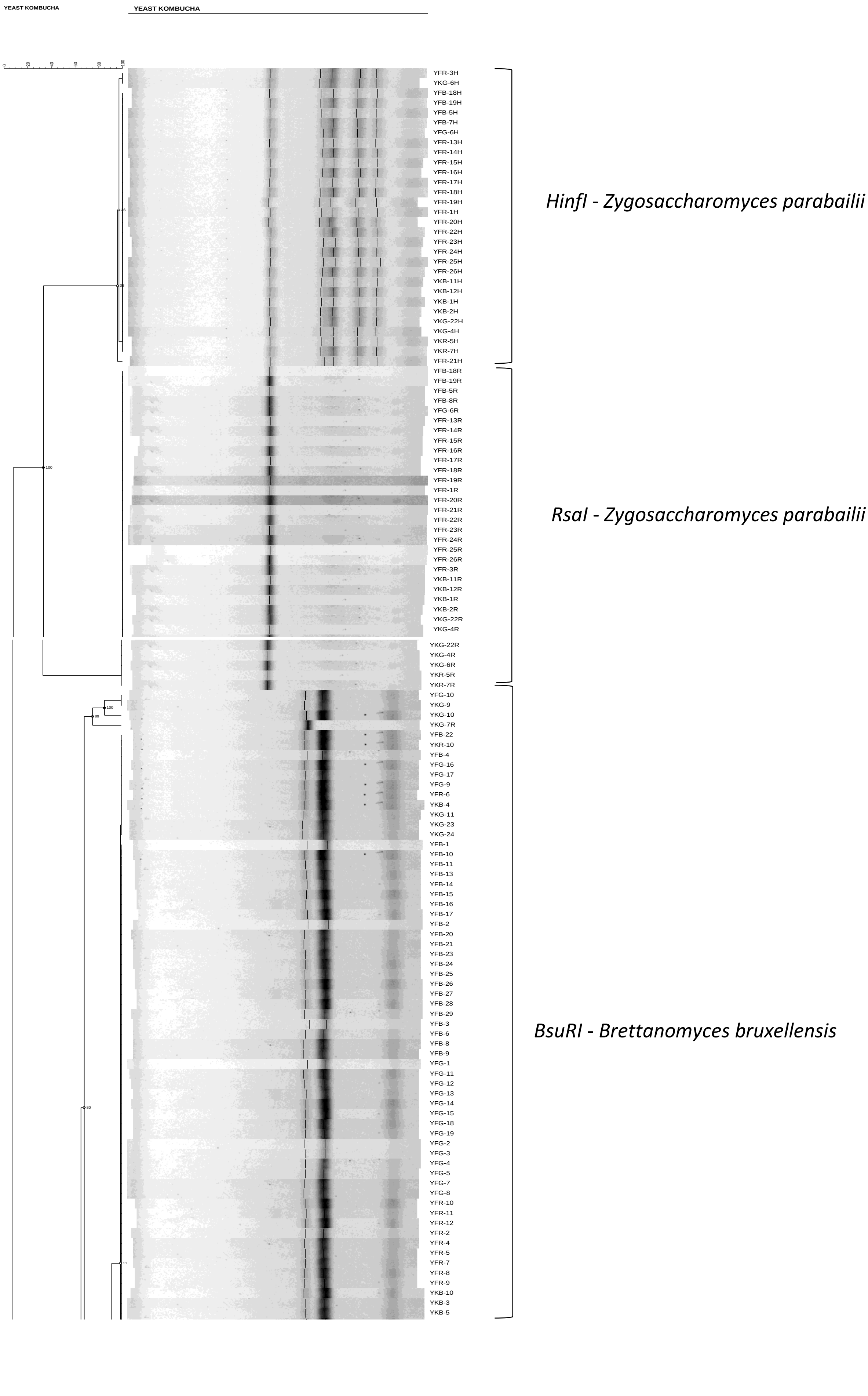
YEAST KOMBUCHA
YEAST KOMBUCHA
0
20
40
60
80
100
100
100
89
80
YFR-3H
YKG-6H
YFB-18H
YFB-19H
YFB-5H
YFB-7H
YFG-6H
YFR-13H
YFR-14H
YFR-15H
YFR-16H
YFR-17H
YFR-18H
YFR-19H
YFR-1H
YFR-20H
YFR-22H
YFR-23H
YFR-24H
YFR-25H
YFR-26H
YKB-11H
YKB-12H
YKB-1H
YKB-2H
YKG-22H
YKG-4H
YKR-5H
YKR-7H
YFR-21H
YFB-18R
YFB-19R
YFB-5R
YFB-8R
YFG-6R
YFR-13R
YFR-14R
YFR-15R
YFR-16R
YFR-17R
YFR-18R
YFR-19R
YFR-1R
YFR-20R
YFR-21R
YFR-22R
YFR-23R
YFR-24R
YFR-25R
YFR-26R
YFR-3R
YKB-11R
YKB-12R
YKB-1R
YKB-2R
YKG-22R
YKG-4R
YKG-22R
YKG-4R
YKG-6R
YKR-5R
YKR-7R
YFG-10
YKG-9
YKG-10
YKG-7R
YFB-22
YKR-10
YFB-4
YFG-16
YFG-17
YFG-9
YFR-6
YKB-4
YKG-11
YKG-23
YKG-24
YFB-1
YFB-10
YFB-11
YFB-13
YFB-14
YFB-15
YFB-16
YFB-17
YFB-2
YFB-20
YFB-21
YFB-23
YFB-24
YFB-25
YFB-26
YFB-27
YFB-28
YFB-29
YFB-3
YFB-6
YFB-8
YFB-9
YFG-1
YFG-11
YFG-12
YFG-13
YFG-14
YFG-15
YFG-18
YFG-19
YFG-2
YFG-3
YFG-4
YFG-5
YFG-7
YFG-8
YFR-10
YFR-11
YFR-12
YFR-2
YFR-4
YFR-5
YFR-7
YFR-8
YFR-9
YKB-10
YKB-3
YKB-5
HinfI - Zygosaccharomyces parabailii
RsaI - Zygosaccharomyces parabailii
BsuRI - Brettanomyces bruxellensis

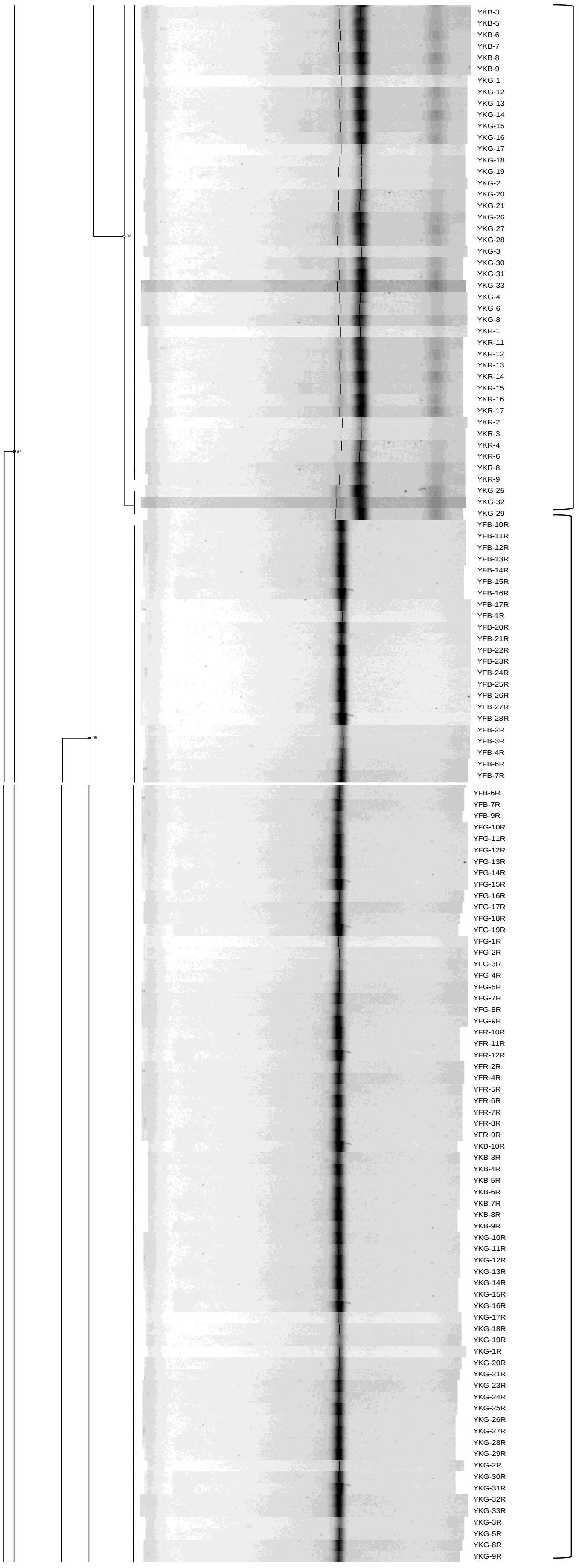

RsaI - Brettanomyces bruxellensis

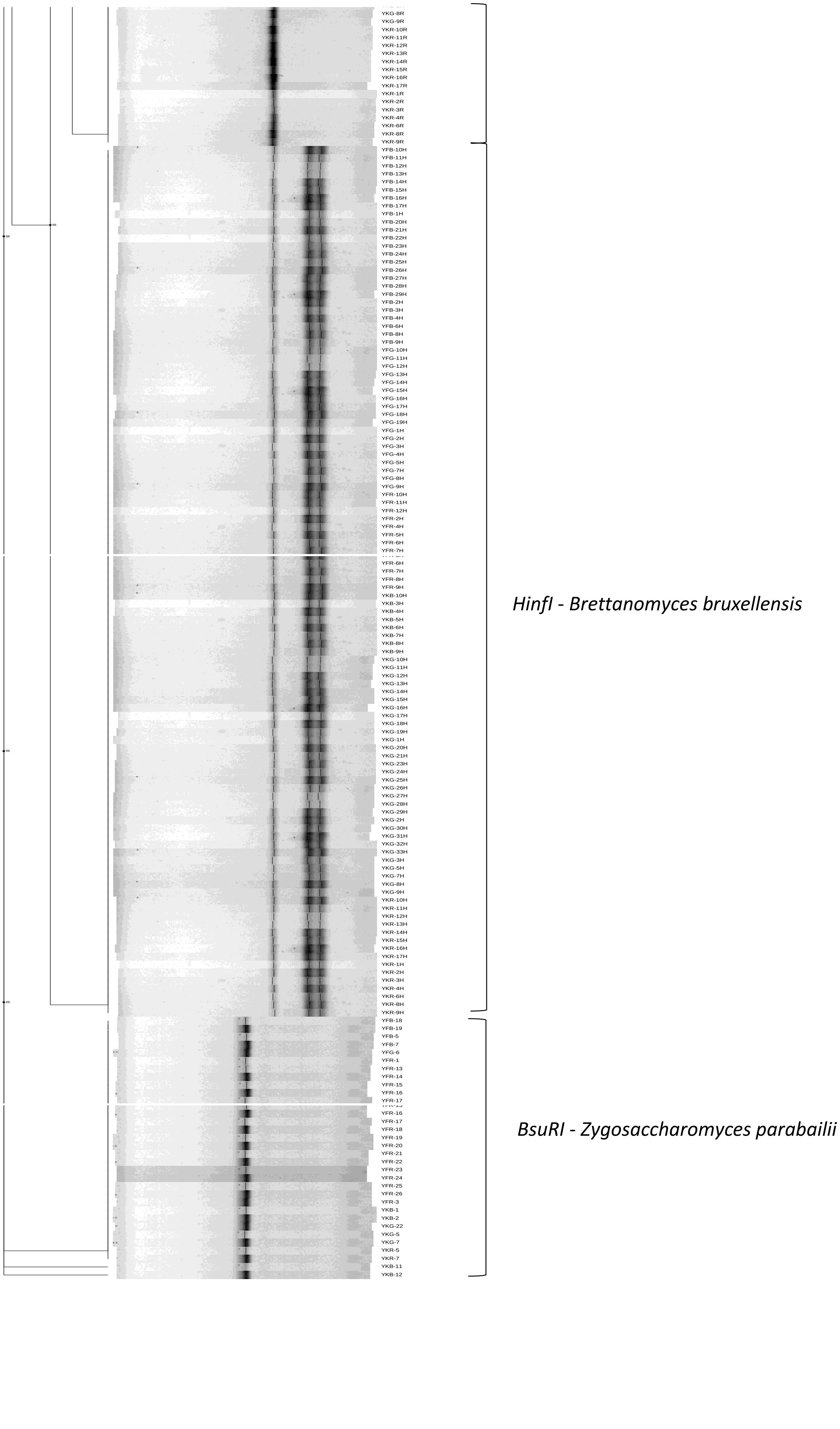
YKG-8R
YKG-9R
YKR-10R
YKR-11R
YKR-12R
YKR-13R
YKR-14R
YKR-15R
YKR-16R
YKR-17R
YKR-1R
YKR-2R
YKR-3R
YKR-4R
YKR-6R
YKR-8R
YKR-9R
YFB-10H
YFB-11H
YFB-12H
YFB-13H
YFB-14H
YFB-15H
YFB-16H
YFB-17H
YFB-1H
YFB-20H
YFB-21H
YFB-22H
YFB-23H
YFB-24H
YFB-25H
YFB-26H
YFB-27H
YFB-28H
YFB-29H
YFB-2H
YFB-3H
YFB-4H
YFB-6H
YFB-8H
YFB-9H
YFG-10H
YFG-11H
YFG-12H
YFG-13H
YFG-14H
YFG-15H
YFG-16H
YFG-17H
YFG-18H
YFG-19H
YFG-1H
YFG-2H
YFG-3H
YFG-4H
YFG-5H
YFG-7H
YFG-8H
YFG-9H
YFR-10H
YFR-11H
YFR-12H
YFR-2H
YFR-4H
YFR-5H
YFR-6H
YFR-7H
YFR-6H
YFR-7H
YFR-8H
YFR-9H
YKB-10H
YKB-3H
YKB-4H
YKB-5H
YKB-6H
YKB-7H
YKB-8H
YKB-9H
YKG-10H
YKG-11H
YKG-12H
YKG-13H
YKG-14H
YKG-15H
YKG-16H
YKG-17H
YKG-18H
YKG-19H
YKG-1H
YKG-20H
YKG-21H
YKG-23H
YKG-24H
YKG-25H
YKG-26H
YKG-27H
YKG-28H
YKG-29H
YKG-2H
YKG-30H
YKG-31H
YKG-32H
YKG-33H
YKG-3H
YKG-5H
YKG-7H
YKG-8H
YKG-9H
YKR-10H
YKR-11H
YKR-12H
YKR-13H
YKR-14H
YKR-15H
YKR-16H
YKR-17H
YKR-1H
YKR-2H
YKR-3H
YKR-4H
YKR-6H
YKR-8H
YKR-9H
YFB-18
YFB-19
YFB-5
YFB-7
YFG-6
YFR-1
YFR-13
YFR-14
YFR-15
YFR-16
YFR-17
YFR-16
YFR-17
YFR-18
YFR-19
YFR-20
YFR-21
YFR-22
YFR-23
YFR-24
YFR-25
YFR-26
YFR-3
YKB-1
YKB-2
YKG-22
YKG-5
YKG-7
YKR-5
YKR-7
YKB-11
YKB-12
HinfI - Brettanomyces bruxellensis
BsuRI - Zygosaccharomyces parabailii

Figure S2. **–** Cluster analysis of RFLP yeasts isolates in kombucha beverage and biofilm obtained using Gel Compare 6.6 (Applied Maths, Sint-Martens-Latem, Belgium). with the Dice's Coefficient of similarity and the un-weighted pair group method arithmetic averages clustering algorithm
