# Supplementary material for: Kombucha Beverage from Green, Black and Rooibos Teas: A Comparative Study Looking at Microbiology, Chemistry and Antioxidant Activity
Source: Nutrients. 2018 Dec 20;11(1):1. doi: 10.3390/nu11010001 (PMC6356548; doi:10.3390/nu11010001)
Supplement: Supplementary file 1 [file nutrients-11-00001-s001.zip › Nuova cartella/Suppl_Mat_Nutrients_final.pdf]

Table S1. Lactobacilli (LAB) and yeasts count on films and kombuchas at 7 and 14 days of fermentation. Different letters are significantly different ($P \leq 0.05$); N.D= not detected

Substrate	Sample	LAB (log_{10} cfu/mL)	Yeasts (log_{10} cfu/mL)
Green Tea	F_{14} G	N.D	7.97±0.08 [a]
	K_{14} G	N.D	7.49±0.09 [b]
Black Tea	F_{14} B	N.D	6.83±0.02 [c]
	K_{14} B	N.D	6.91±0.06 [c]
Rooibois	F_{14} R	N.D	7.08±0.01 [c]
	K_{14} R	N.D	7.36±0.01 [b]

Table S2. Identification and grouping of isolated AAB strains from biofilms and kombucha obtained from fermentation of black. green and rooibos teas.

Isolates	ID (% similarity)	Cluster	Isolates
FR-3; FR-10	*Komagateibacter* spp. (99%)	1	FR 1.2.3.4.5.6.7.9.10.11.12.13.15.17<br>KB 26.27<br>KG 20.21.22.23.24.25<br>KR 18.19.20.23
FG-14	*Komagateibacter* spp. (99%)	2	FB4. FB5. FG14
FB-12<br>KB-16	*K. intermedius* (100%)	3	FB 3.4.5.7.11.12.13<br>FG 14<br>FR 16<br>KB 1.2.3.4.7.15.16<br>KG 19. 20<br>KR 21.22.24
KG-15	*Komagateibacter* spp. (99%)	4	FB 1<br>FG 1.2.3.4.5.6.7.8.9<br>KB 10.11.12.14<br>KG 15
KR-9	*K. intermedius* (100%)	5	FB 6.8.10<br>KR 7. 9
KR-17	*K. rhaeticus* (100%)	6	FB 2<br>FG 10.11.12.13.15<br>FR 8<br>KB 5.6.13<br>KG 3.6.8<br>KR 12. 15. 17
KR-11	*G. entanii* (100%)	7	KR 6.8.11.13.14
KR-1<br>KR-3	*G. entanii* (100%)	8	KR-1; KR-3
KR-2<br>KR-4<br>KR-5<br>KG-16	*K. intermedius* (100%)	8	KR-2; KR-4; KR-5; KG.16
KG-2<br>KG-5	*K. intermedius* (100%)	9	KG 1. 2. 4. 5. 7. 9. 10. 11. 12
KG-13	*G. entanii* (100%)	10	KG-13
KB-17	*K. intermedius* (100%)	11	KB 17. 18. 19. 20. 21. 22. 23. 24. 25

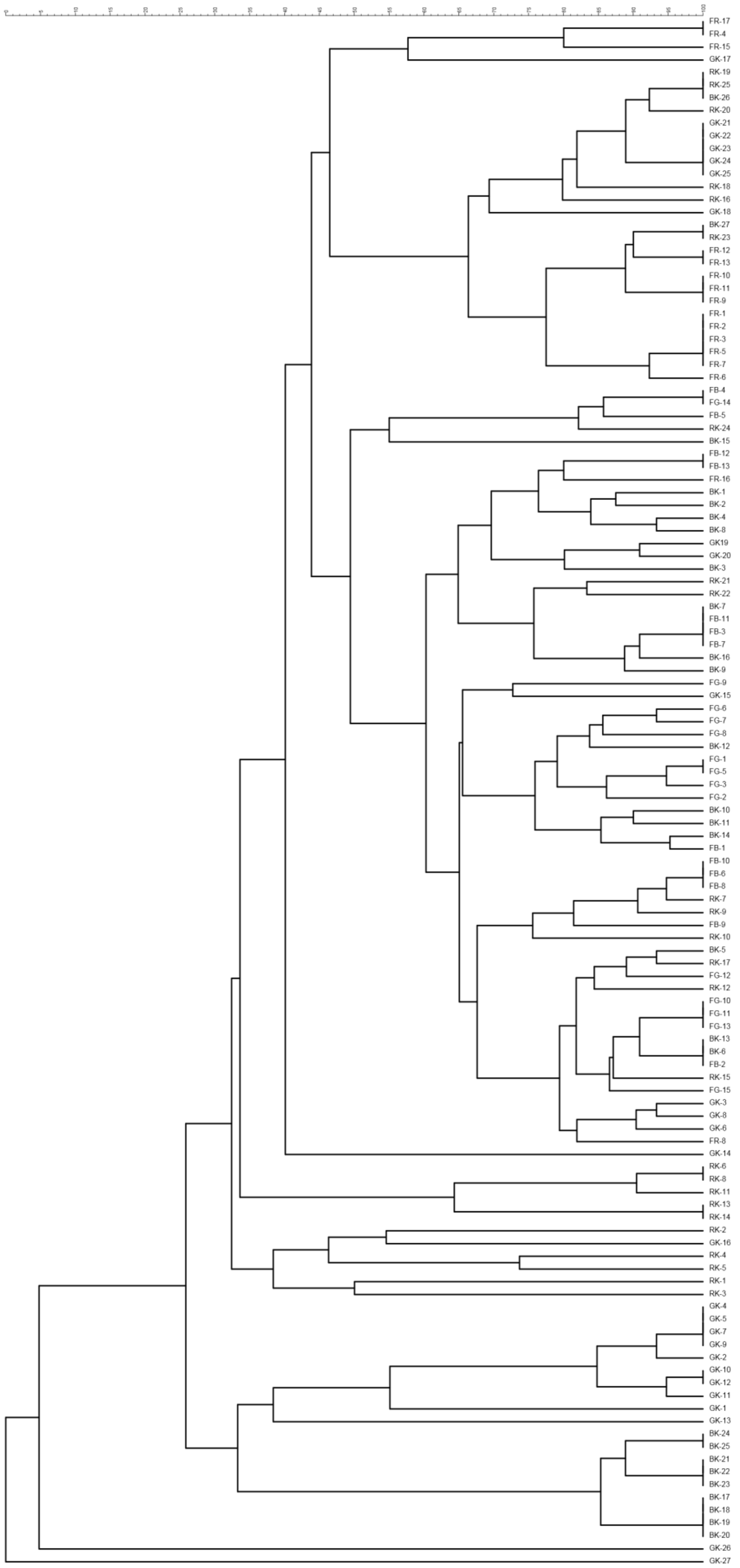
FR-17
FR-4
FR-15
GK-17
RK-19
RK-25
BK-26
RK-20
GK-21
GK-22
GK-23
GK-24
GK-25
RK-18
RK-16
GK-18
BK-27
RK-23
FR-12
FR-13
FR-10
FR-11
FR-9
FR-1
FR-2
FR-3
FR-5
FR-7
FR-6
FB-4
FG-14
FB-5
RK-24
BK-15
FB-12
FB-13
FR-16
BK-1
BK-2
BK-4
BK-8
GK19
GK-20
BK-3
RK-21
RK-22
BK-7
FB-11
FB-3
FB-7
BK-16
BK-9
FG-9
GK-15
FG-6
FG-7
FG-8
BK-12
FG-1
FG-5
FG-3
FG-2
BK-10
BK-11
BK-14
FB-1
FB-10
FB-6
FB-8
RK-7
RK-9
FB-9
RK-10
BK-5
RK-17
FG-12
RK-12
FG-10
FG-11
FG-13
BK-13
BK-6
FB-2
RK-15
FG-15
GK-3
GK-8
GK-6
FR-8
GK-14
RK-6
RK-8
RK-11
RK-13
RK-14
RK-2
GK-16
RK-4
RK-5
RK-1
RK-3
GK-4
GK-5
GK-7
GK-9
GK-2
GK-10
GK-12
GK-11
GK-1
GK-13
BK-24
BK-25
BK-21
BK-22
BK-23
BK-17
BK-18
BK-19
BK-20
GK-26
GK-27

Figure S1. Cluster analysis of the AAB profiles obtained by RAPD-PCR. using Bionumerics 7.1 (Applied Maths. Sint-Martens-Latem. Belgium) with the Dice's Coefficient of similarity and the un-weighted pair group method arithmetic averages clustering algorithm (UPGMA).

a

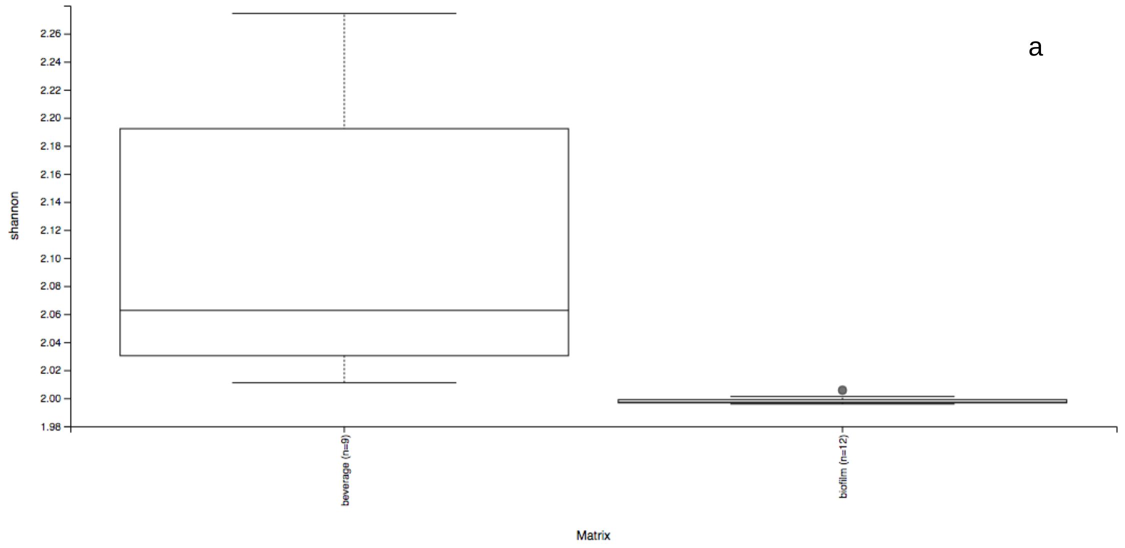

Kruskal-Wallis (pairwise)

Download CSV

Group 1	Group 2	H	p-value	q-value
beverage (n=9)	biofilm (n=12)	14.727273	0.000124	0.000124

b

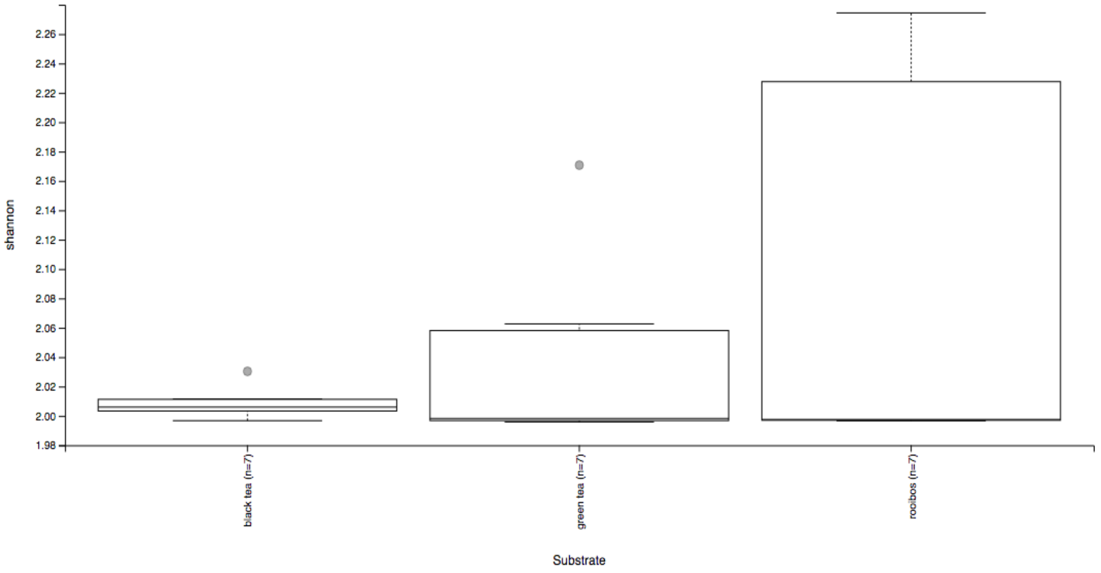

Kruskal-Wallis (pairwise)

Download CSV

Group 1	Group 2	H	p-value	q-value
black tea (n=7)	green tea (n=7)	0.036735	0.848006	0.848006
	rooibos (n=7)	0.036735	0.848006	0.848006
green tea (n=7)	rooibos (n=7)	0.330612	0.565299	0.848006

Figure S3. Alpha diversity of bacterial Component a) Shannon diversity by matrix; b) Shannon diversity by substrate) with Kruskal-Wallis pairwise analysis

a

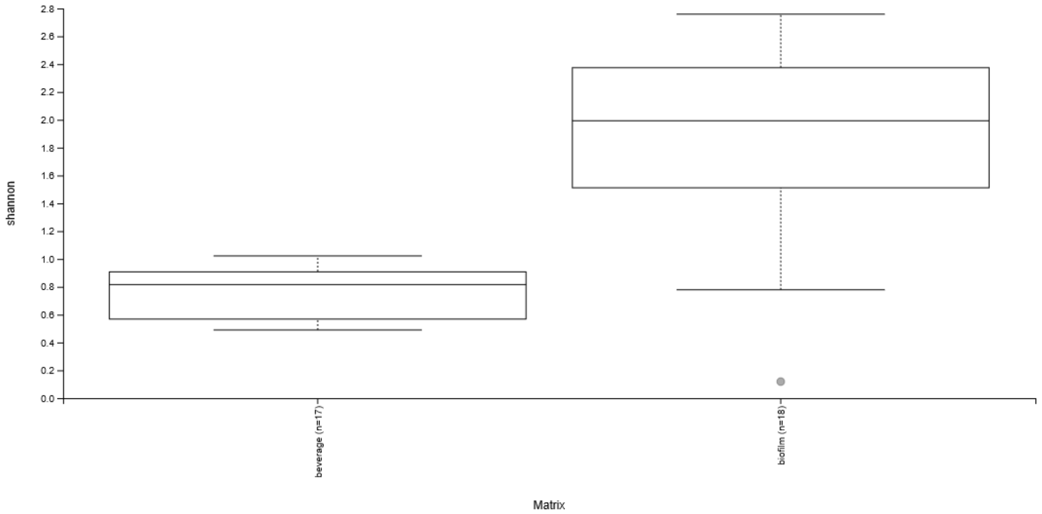

Kruskal-Wallis (pairwise)

Download CSV

Group 1	Group 2	H	p-value	q-value
**beverage (n=17)**	**biofilm (n=18)**	17.569717	0.000028	0.000028

b

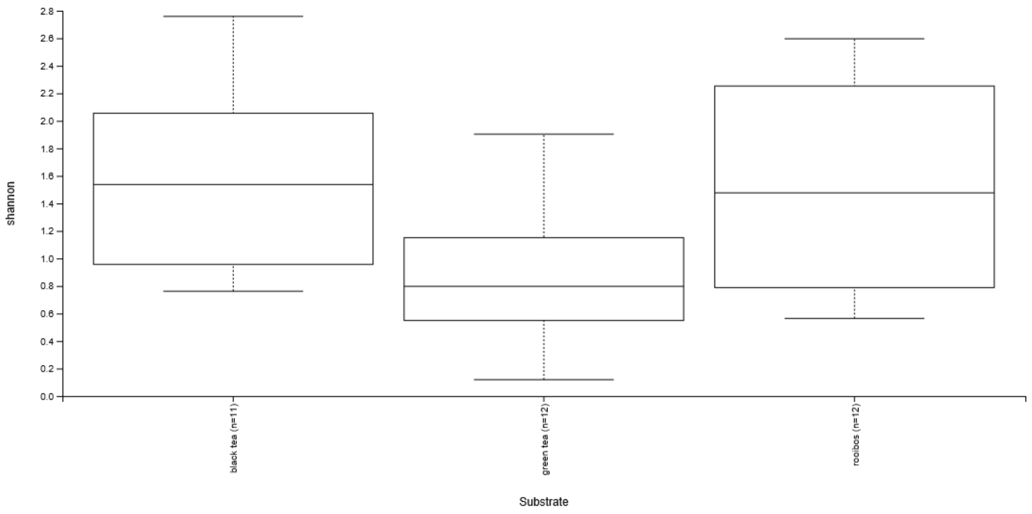

Kruskal-Wallis (pairwise)

Download CSV

Group 1	Group 2	H	p-value	q-value
black tea (n=11)	green tea (n=12)	5.761364	0.016382	0.049147
	rooibos (n=12)	0.185606	0.666599	0.666599
green tea (n=12)	rooibos (n=12)	3.853333	0.049647	0.074471

c

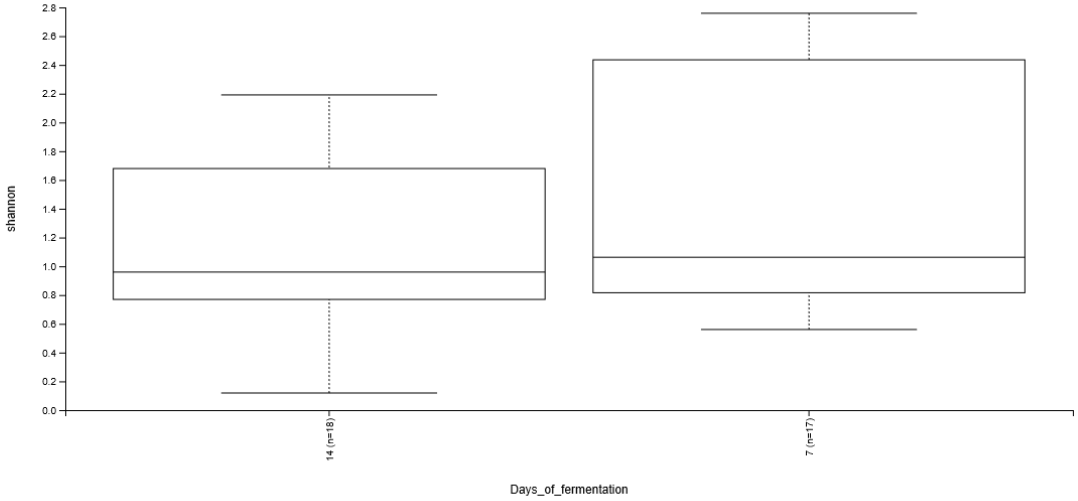

Kruskal-Wallis (pairwise)

Download CSV

Group 1	Group 2	H	p-value	q-value
14 (n=18)	7 (n=17)	1.046841	0.306236	0.306236

Figure S4. Alpha diversity of fungal component a) Shannon diversity by matrix; b) Shannon diversity by matrix; c) Shannon diversity by days of fermentation with the associated Kruskal-Wallis pairwise analysis

Table S3. Relative abundance of bacterial family in beverage and biofilm at 14 days (data expressed as % value)

	$K_{14}B$	$K_{14}G$	$K_{14}R$	$F_{14}B$	$F_{14}G$	$F_{14}R$
**Bifidobacteriaceae**	0.0204	0.0669	0.2648	0.0098	0.0052	0.0032
**Corynebacteriaceae**	0.0016	0.0046	0.0282	0.0015	-	-
**Propionibacteriaceae**	0.0081	0.0416	0.0825	0.0030	-	0.0024
**Coriobacteriaceae**	-	-	0.0049	-	-	-
**Bacteroidaceae**	0.0101	0.0149	0.1134	0.0019	-	0.0021
**Odoribacteraceae**	-	-	0.0058	-	-	-
**Rikenellaceae**	0.0060	0.0135	0.1095	0.0010	-	-
**Paenibacillaceae**	0.0080	0.0160	0.1150	-	-	-
**Staphylococcaceae**	0.0013	0.0039	0.0216	0.0010	-	-
**Lactobacillaceae**	0.0023	0.0067	0.0562	-	-	-
**Leuconostocaceae**	-	0.0020	0.0411	-	-	-
**Streptococcaceae**	0.0023	0.0087	0.0973	-	-	-
**Christensenellaceae**	-	-	0.0046	-	-	-
**Lachnospiraceae**	0.0059	0.0213	0.2208	0.0010	-	-
**Clostridiales family XIII incertae sedis**	-	-	0.0029	-	-	-
**Peptostreptococcaceae**	0.0021	0.0011	0.0023	-	-	-
**Methylobacteriaceae**	0.0019	0.0215	0.0029	-	-	-
**Rhizobiaceae**	-	0.0016	0.0054	-	-	-
**Rhodobacteraceae**	-	0.0015	0.0133	-	-	-
**Acetobacteraceae**	99.826	97.689	98.034	99.956	99.983	99.977
**Sphingomonadaceae**	-	0.0077	0.0222	-	-	-
**Comamonadaceae**	0.0020	0.0067	0.0076	-	-	-
**Enterobacteriaceae**	0.0092	0.0442	0.2627	0.0045	0.0025	0.0022
**Moraxellaceae**	-	0.0022	0.0021	-	-	-
**Akkermansiaceae**	0.0010	0.0052	0.0378	0.0013	-	-
**Others**	0.0036	0.0034	0.0055	0.0028	0.0035	0.0040

Table S4. - Relative abundance of yeasts family in biofilm at 14 days (data expressed as % value)

	F_7B	F_7G	F_7R	$F_{14}B$	$F_{14}G$	$F_{14}R$
**Pichiaceae**	60.9953	85.8813	40.0155	77.1077	92.2394	60.1906
**Saccharomycetaceae**	38.9819	14.0579	57.9034	22.8143	7.4258	31.8865
**Metschnikowiaceae**	0.0038	0.0037	0.4743	-	0.0024	0.0017
**Sporidiobolaceae**	-	-	0.0269	-	-	0.0112
**Malasseziaceae**	-	-	0.0318		0.0079	0.0900
**Pleosporaceae**	-	0.0071	-	0.0466	-	0.0012
**Aspergillaceae**	0.0049	-	0.2875	0.0231	0.1565	0.3238
**Debaryomycetaceae**	-	0.0028	-	-	-	-
**Dipodascaceae**	-	-	-	-	-	-
**Phaffomycetaceae**	-	-	-	-	-	-
**Cordycipitaceae**	-	-	0.0144	-	-	-
**Schizoporaceae**	-	-	0.1019	-	-	-
**Coriolaceae**	-	-	0.0000	-	0.0026	-
**Meruliaceae**	-	-	0.0163	-	0.0012	6.7560
**Mrakiaceae**	-	-	-	-	-	0.0139
**Tremellaceae**	-	-	-	-	-	-
**Trichosporonaceae**	-	-	-	-	-	-
**Others**	0.0078	0.0118	1.0341	0.0297	0.1633	0.7250

Table S5. Relative abundance of yeasts family in beverage at 14 days (data expressed as % value)

	K_7B	K_7G	K_7R	$K_{14}B$	$K_{14}G$	$K_{14}R$
**Pichiaceae**	91.3660	93.5626	93.3040	91.7686	95.7010	91.7128
**Saccharomycetaceae**	8.5638	6.2313	5.8145	8.2071	4.2126	8.1625
**Metschnikowiaceae**	0.0135	0.0892	0.0433	0.0029	0.0452	0.0131
**Sporidiobolaceae**	0.0054	-	-	-	-	-
**Malasseziaceae**	0.0039	-	0.0040	-	0.0013	-
**Pleosporaceae**	-	0.0052	0.0762	-	0.0081	-
**Aspergillaceae**	-	0.0028	0.0000	0.0009	0.0005	-
**Debaryomycetaceae**	-	0.0102	0.0214	-	0.0022	-
**Dipodascaceae**	-	-	0.1646	-	-	-
**Phaffomycetaceae**	-	0.0298	0.1174	-	-	0.0052
**Cordycipitaceae**	-	-	-	-	-	-
**Schizoporaceae**	-	-	-	-	-	-
**Coriolaceae**	-	-	0.0363	-	-	-
**Meruliaceae**	-	-	-	0.0017	-	0.0167
**Mrakiaceae**	-	-	-	-	-	-
**Tremellaceae**	-	-	-	-	-	0.0055
**Trichosporonaceae**	-	0.0048	0.0854	-	-	0.0023
**Others**	0.0388	0.0641	0.3506	0.0109	0.0288	0.0474

Table S6. Pairwise Kruskall-Wallis comparisons of alpha diversity of fungal component by Shannon index at 7 and 14 days separate

**7 days samples**				
**Group 1**	**Group 2**	**H**	**p-value**	**q-value**
Kombucha (n=8)	Biofilm (n=9)	12	0.00053201	0.00053201
KB (n=5)	KG (n=6)	1.633333333	0.20124262	0.60372786
KB (n=5)	KR (n=6)	0.133333333	0.71500065	0.71500065
KG (n=6)	KR (n=6)	0.41025641	0.52183939	0.71500065
**14 days samples**				
**Group 1**	**Group 2**	**H**	**p-value**	**q-value**
Kombucha (n=9)	Biofilm (n=9)	5.475633528	0.01928337	0.01928337
KB (n=6)	KG (n=6)	5.76923077	0.01630917	0.04892752
KB (n=6)	KR (n=6)	0	1	1
KG (n=6)	KR (n=6)	4.33333333	0.03737299	0.05605948

Table S7. Beta diversity – Fungal parwisise ANOSIM by Bray-Curtis Dissimilarity. at 7 and 14 days separate

**7 days samples**						
Group 1	Group 2	Sample size	Permutations	R	p-value	q-value
Kombucha	Biofilm	17	999	0.25998264	0.011	0.011
KB	KG	11	999	0.336	0.034	0.051
KB	KR	11	999	0.39466667	0.028	0.051
KG	KR	12	999	0.24814815	0.066	0.066
**14 days samples**						
Group 1	Group 2	Sample size	Permutations	R	p-value	q-value
Kombucha	Biofilm	18	999	0.06824417	0.064	0.064
KB	KG	12	999	0.21296296	0.004	0.006
KB	KR	12	999	0.28148148	0.003	0.006
KG	KR	12	999	0.18148148	0.024	0.024
